# Longevity GWAS Using the *Drosophila* Genetic Reference Panel

**DOI:** 10.1093/gerona/glv047

**Published:** 2015-04-28

**Authors:** Dobril K. Ivanov, Valentina Escott-Price, Matthias Ziehm, Michael M. Magwire, Trudy F. C. Mackay, Linda Partridge, Janet M. Thornton

**Affiliations:** ^1^European Molecular Biology Laboratory, European Bioinformatics Institute (EMBL-EBI), Wellcome Trust Genome Campus, Hinxton, Cambridge, UK.; ^2^Medical Research Council Centre for Neuropsychiatric Genetics and Genomics, Institute of Psychological Medicine and Clinical Neurosciences, Cardiff University, UK.; ^3^Department of Genetics Evolution and Environment, The Institute of Healthy Ageing, University College London, UK.; ^4^Department of Biological Sciences, Program in Genetics and W. M. Keck Center for Behavioral Biology, North Carolina State University, Raleigh.; ^5^Syngenta, Research Triangle Park, North Carolina.; ^6^Max Planck Institute for Biology of Ageing, Cologne, Germany.

**Keywords:** Ageing, Gene-based analysis, Gene ontology, Polygenic score analysis, Target of rapamycin, Insulin signaling pathway

## Abstract

We used 197 *Drosophila melanogaster* Genetic Reference Panel (DGRP) lines to perform a genome-wide association analysis for virgin female lifespan, using ~2M common single nucleotide polymorphisms (SNPs). We found considerable genetic variation in lifespan in the DGRP, with a broad-sense heritability of 0.413. There was little power to detect signals at a genome-wide level in single-SNP and gene-based analyses. Polygenic score analysis revealed that a small proportion of the variation in lifespan (~4.7%) was explicable in terms of additive effects of common SNPs (≥2% minor allele frequency). However, several of the top associated genes are involved in the processes previously shown to impact ageing (eg, carbohydrate-related metabolism, regulation of cell death, proteolysis). Other top-ranked genes are of unknown function and provide promising candidates for experimental examination. Genes in the target of rapamycin pathway (TOR; *Chrb*, *slif*, *mipp2*, *dredd*, *RpS9*, *dm*) contributed to the significant enrichment of this pathway among the top-ranked 100 genes (*p* = 4.79×10^−06^). Gene Ontology analysis suggested that genes involved in carbohydrate metabolism are important for lifespan; including the InterPro term DUF227, which has been previously associated with lifespan determination. This analysis suggests that our understanding of the genetic basis of natural variation in lifespan from induced mutations is incomplete.

Our understanding of the genetic factors affecting longevity has come primarily from experimental work with model organisms. Single-gene mutations in several biological pathways in a range of model organisms can significantly increase lifespan. Among others, perturbations in the nutrient-sensing pathways, the target of rapamycin (TOR; 1–2) and the insulin/insulin-like growth factor (IIS) signaling pathways, can ameliorate the ageing process in worms, flies, and mice (3–5). In model organisms, mutations include both knock-outs and strong over-expression, which can give rise to large effects on lifespan. However, mutations with these large effects are unlikely to be segregating in natural populations (including human populations). It is well established that there is a genetic component for survival into old age in humans. Estimates of heritability from twin-registry and population-based studies range from ~20% to ~30% (6–8), and can increase with age (7). However, the paucity of gene variants found by genome-wide association studies (GWAS) of lifespan in humans suggests that effects of individual genetic variants are small. Thus far, the most robustly replicated finding is the importance of the *APOE* gene (6,9–11), where the ε4 isoform is repeatedly associated with increased mortality in carriers (12). Therefore, *APOE* is considered to be a “frailty gene,” rather than a “longevity gene” (6,12). GWAS have also implicated several other genes associated with longevity, although none reach genome-wide significance (reviewed in 6). In addition to GWAS, association analyses of human orthologs of candidate longevity genes found in model organisms have been performed (13–15). These studies have identified genes in the IIS signaling pathway as being associated with human longevity. Indeed, the human forkhead box O3 transcription factor (*FOXO3*) in this pathway is often described as the second gene most commonly associated with longevity (15–17), although these findings have not been replicated in several other studies (9,10,18,19).

Thus, studying natural variation in longevity in animal models could bridge the gap between mutations with a large effect, found in model organisms, and natural variants in humans. A substantial difference between studies in humans and those in animal models is the controlled environment in which the latter are living. This reduces the impact of environmental factors, allowing a larger proportion of differences in the phenotypic variation to be attributed to genetic variation.

Genome-wide association studies of ageing in model organisms have rarely been performed. The *Drosophila melanogaster* Genetic Reference Panel (DGRP) of ~200 inbred, sequenced lines is a community resource for analysis of population genomics and genome-wide association mapping of quantitative traits (20,21). Here, we performed a GWAS of virgin female lifespan using 197 DGRP lines (for a list of the lines used and their mean lifespan see Supplementary Table 1). Our aim was to measure the influence of naturally occurring genetic variants on lifespan to discover genes and mechanisms associated with natural variation in lifespan. We utilized an unbiased, multilayered approach building on a single-single nucleotide polymorphism (SNP) GWAS as a primary unit, and tested whether individual genes, including regulatory regions, are associated with lifespan. We also explored whether enrichment of particular domains and/or biological pathways contribute to an increased or reduced mean lifespan. In addition, we investigated how much of the phenotypic variance in lifespan is explicable in terms of the additive effects of common genetic variants.

## Materials and Methods

### Lines Used

The *Drosophila* Genetic Reference Panel, Freeze 2.0 (20,21), comprises 205 *D. melanogaster* lines derived by 20 generations of full-sib mating from inseminated wild-type caught females from Raleigh, North Carolina. Whole-genome sequencing data, along with genotype calls, are available for all 205 lines (http://dgrp2.gnets.ncsu.edu). Lifespan data were obtained for virgin females for 197 DGRP lines, with a sample size of *N* = 25 females per line (five females in each of five replicate vials). A subset of the lifespan data has been published previously (22). The *Wolbachia* infection status and karyotype for major inversions for each line were downloaded from the DGRP website.

### Quality Control

The original number of DGRP SNPs, after removing nonpolymorphic and SNPs with ≥ 3 alleles, was 3,963,420. We further filtered these data. All SNPs from the different chromosomes (ie, *2L*/*2R*, *3L*/*3R*, *4*, and *X*) were combined before performing these filters. We set the SNP call rate cut-off at 0.9 (Supplementary Figure 1), that is, 90% of the lines were required to have a genotype call for a particular SNP to be included for further analysis. The minor allele frequency (MAF) cut-off was set at 0.02 (Supplementary Figure 2), that is, SNPs that had MAF <2% were excluded from further analysis. Finally, at least 80% of the SNPs were present in all individuals (Supplementary Figures 3 and 4), thus no individual lines were removed. A total of 2,193,745 SNPs remained for 197 DGRP lines after applying these filters (summary statistics are presented in Supplementary Table 1).

### Single-SNP Association (GWAS) Analysis

In order to identify SNPs associated with lifespan in the DGRP inbred lines, linear regression under an additive model was used, with a covariate included for the presence of *Wolbachia pipientis* and the first four principal components [using Eigenstrat software (23)]. For the calculations, we used PLINK v. 1.07 (24). All single SNP association results are available upon request.

### Gene-Based Association Analysis

We assigned the 2,193,745 SNPs to genes (including intronic regions) (Ensembl; http://www.ensembl.org/biomart/martview; BDGP5; as defined by the “Gene start (bp)” and “Gene end (bp)” in biomart). In some cases, SNPs were assigned to multiple genes, that is, when genes overlapped. We used a previously described, set-based method (25), whereby *p* values from all SNPs within a gene from the single-SNP association, corrected for *Wolbachia* status and the first four principal components, were combined using Fisher’s *T*-statistic:

$$T=-2\sum_{i=1}^{N}\log_{e}p_{i}$$

where *N* is the number of SNPs (tests) and *p*_*i*_ (*i* = 1 ,…, *N*) is the corresponding *p* value. For each gene, this was termed original statistic. Only genes with at least two SNPs were used in the calculations of the gene-based analysis. To calculate empirical gene-wide significance for each gene, we performed 10,000 genome-wide permutations (with replacement) for each set of SNPs within a gene. For each gene in each permutation, we calculated a permutated Fisher’s *T*-statistic and compared it with the original statistic. The empirical *p* value obtained for each gene was the proportion of permuted Fisher’s *T*-statistic that was found equal or larger to the original statistic. For the top 40 genes we performed 1,000,000 permutations, in order to get a more accurate estimate of the empirical gene-significance. All gene-based results are available upon request. We also performed similar gene-based analysis for SNPs within ± 5kb (kilobases) of each gene.

### Polygenic Score Analysis

Testing the polygenic score association (as described in the International Schizophrenia Consortium (ISC) study, 26) with lifespan is based on the notion that a phenotype, under the polygenic model, is affected/influenced by the combined effect from numerous variants, each with a small effect. Such variants would not generally be significant in a single-SNP analysis. Nevertheless, such truly associated variants would be clustered in the lower percentile of the distribution of the association of SNPs. Hence, polygenic scores, created by using weighted allele dosage of SNPs passing a certain permissive *p* value threshold, could explain a relatively large proportion of the phenotypic variance. An independent set of lines with recorded lifespan was not available in order to calculate polygenic scores on the DGRP sample (discovery set) and test in a test sample set (target set), as done in the ISC. Instead, we performed a cross-validation analysis using permutations (with replacement). The DGRP sample set was randomly split in two sets (discovery and target sample sets) with roughly equal number of lines (99 vs 98). As in the ISC study, we pruned (maximum linkage-disequilibrium: *r* ^2^ = 0.25) the SNPs present in each of the discovery sample sets (99 lines) within a sliding window of 250kb and kept the highest associated SNP (using clump function in PLINK v. 1.07). We utilized 14 separate *p* value thresholds (*p*_*t*_ ≤ 1.0, *p*_*t*_ ≤ 0.9, *p*_*t*_ ≤ 0.8, *p*_*t*_ ≤ 0.7, *p*_*t*_ ≤ 0.6, *p*_*t*_ ≤ 0.5, *p*_*t*_ ≤ 0.2, *p*_*t*_ ≤ 0.1, *p*_*t*_ ≤ 0.05, *p*_*t*_ ≤ 0.01, *p*_*t*_ ≤ 0.005, *p*_*t*_ ≤ 0.001, *p*_*t*_ ≤ 0.0005, *p*_*t*_ ≤ 0.0001) for selection of SNPs in the discovery sample set for the calculation of the polygenic scores. The polygenic scores were calculated by using the score function in PLINK, based on the number of alleles from the discovery set that passed a certain *p* value threshold and weighted by the ß-coefficients from the linear regression in the discovery set (including *Wolbachia* status and the first four principal components as covariates). The final variance explained in terms of *R* ^2^ was calculated by means of linear regression of lifespan on polygenic scores. This procedure was repeated 100 times and means and standard deviations for each of the sets were calculated.

### Gene Ontology and InterPro

Based on the ranking of the gene-based association analysis, we performed a Gene Ontology (GO, http://geneontology.org) and InterPro (http://www.ebi.ac.uk/interpro/ (27);) over-representation/enrichment analysis. Conversion from FlyBase gene identifiers to GO and InterPro was achieved by using FlyBase version FB2014_04. The ranked gene list (14,146 genes for SNPs within genes and 15,145 for SNPs within and ±5kb around genes), based on the gene-based *p* values, were tested for GO term over-representation by using the software Catmap (28). Catmap uses a Wilcoxon rank-sum test to assign significance of GO and/or InterPro categories among ranked list of genes.

## Results

### Phenotypic Variation in Lifespan in the DGRP

**Figure 1.**
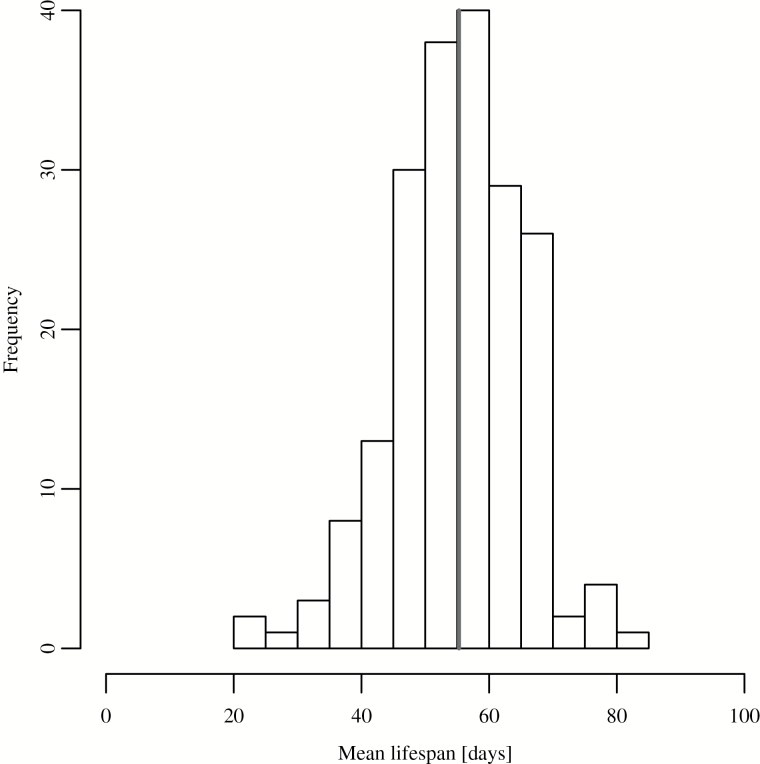
Distribution of lifespan (197 lines). The black vertical line represents the mean lifespan (55.28 days).

The overall mean lifespan for the 197 lines used was 55.28 days (Figure 1). A list of the lines used, along with lifespan data and *Wolbachia* status, can be found in Supplementary Table 1. We found considerable genetic variation in lifespan, with a broad-sense heritability of *H* ^2^ = 0.413 (Figure 1, Supplementary Tables 1 and 2).

There is considerable lifespan variability between the fly lines (Figure 1). The maximal mean lifespan in the DGRP (80.29 days) is similar to the maximal mean lifespan achieved in mutants (long-lived isogenic fly line 1L18; mean lifespan 82.1 (29) and median lifespan 82.5 days for fly strain *w* ^Dah^; *dilp2-3,5* (30)). In addition, the lifespan variability within the fly lines is not that different from a survival analysis of large number of *Drosophila* controls (31). The mean lifespan variability of *Drosophila* strains wDah and w1118 is ~10.37 (data not shown), similar to the variability within the DGRP fly lines (μσ = 10.6; Supplementary Figure 5).

### Analysis of Single-SNP Association with Lifespan

**Figure 2.**
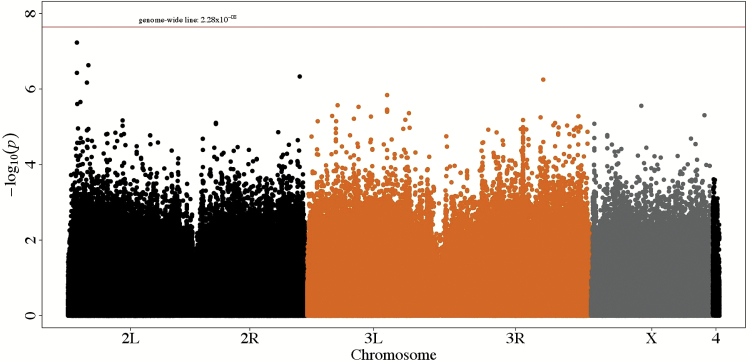
Manhattan plot for single-SNP GWAS. Each point represents a SNP. The height of the SNP represents the strength of association with lifespan, expressed as −log10(*p* value). The red horizontal line represents the genome-wide Bonferroni significance threshold (*p* = 2.28×10^−08^).

Several large polymorphic inversions segregate in the DGRP, and about 50% of the lines are infected with the endosymbiotic bacterium *Wolbachia pipientis* (21). Since both inversion karyotypes and *Wolbachia* infection can cause cryptic relatedness and bias GWAS, we performed a principal component analysis of the DGRP genotypes. We found that *In(3R)Mo* and *In(2L)t* are associated with genetic variation and relatedness within the 197 lines (Supplementary Figure 6). We therefore adjusted the phenotypic data to account for the first four principal components and *Wolbachia* status, although we did not observe a significant effect of *Wolbachia* status on lifespan, and performed a single-variant GWAS for lifespan using the 2,193,745 SNPs with MAF ≥ 0.02. None of the SNPs tested reached the Bonferroni threshold for genome-wide significance (*p* = 2.28×10^−08^; Figure 2 and Supplementary Figure 7). The top 50 SNPs and nearby genes are listed in Supplementary Table 3.

In order to assess whether there was sufficient power to detect a relatively large effect on lifespan, we performed power calculations (Supplementary Figure 8). The power to detect a relatively large effect (10 days difference in lifespan between homozygous genotypes) at α = 2.28×10^−08^ ranged from 15% to 68%, corresponding to MAF of 0.1–0.2 (Supplementary Figure 8). The power increased to >91% power for MAF ≥ 0.3.

Thus, there is enough power to detect common SNPs with large effects on lifespan, indicating that there are no common alleles with large effects segregating in the DGRP. There was also adequate power to detect at least some lower frequency SNPs with large effects, so perhaps these do not segregate in the population either. We therefore infer that the individual variants contributing to the heritability are likely to have effects that are too small to be reliably detected in a population of 197 inbred lines.

Several of the genes tagged by top SNPs have been shown previously to affect lifespan (*brummer*, *Rpd3*, *Thor*, *hairy*, *sima*, and *RFeSP*). Lifespan extension by dietary restriction (DR) is mediated by an increased activity of *Thor* (32), although this may depend on the type of DR, as flies null for *Thor* respond normally to DR (33). *brummer* controls organismal fat storage in *Drosophila* and flies lacking *brummer* under DR live ~56% longer than controls (34). Females heterozygous for a *Rpd3* hypomorphic allele have a lifespan extension of 52% and experimental data suggest that *Rpd3* is likely to be within a pathway related to DR (35).

Both *hairy* and *sima* are involved in cellular responses to hypoxia (36,37), although the relationship between tolerance to hypoxia and lifespan is not entirely clear. That is, flies selected for hypoxia tolerance do not have altered lifespan (36), direct knockdown of genes leading to increased hypoxia resistance in worms also do not affect lifespan (38), although hypoxia significantly induces reactive oxygen species (ROS) within mice adipocytes and tissue hypoxia increases with age (39). Thus, it is unclear if hypoxia tolerance will result in fewer ROS and potentially increased lifespan. Nevertheless, deletions of the *C*. *elegans* ortholog of *sima*, the hypoxia-inducible factor *HIF-1*, modify longevity in a temperature-dependent manner (40). Additionally, the worm ortholog of *Drosophila RFeSP*, *isp-1*, extends lifespan (41).

### Gene-Based Analysis

**Figure 3.**
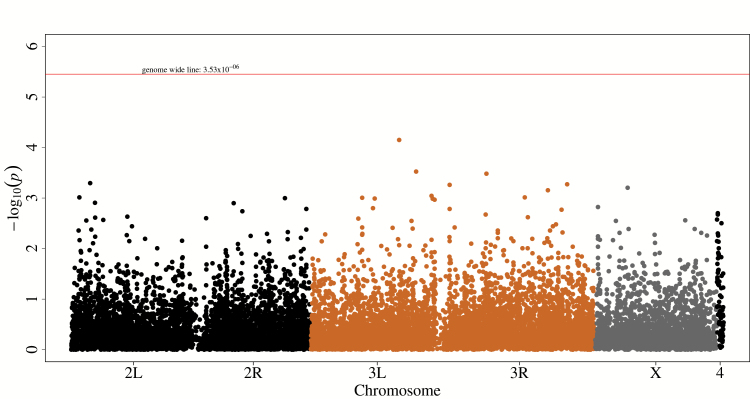
Manhattan plot for gene-based GWAS. Each point represents a gene. The height of the gene represents the strength of association with lifespan, expressed as −log10(*p* value). The red horizontal line represents genome-wide Bonferroni significance threshold (*p* = 3.53×10^−06^).

As noted above, power calculations suggest that single-SNP association analysis is underpowered for SNPs with MAF < 0.3. Gene-based GWAS on the other hand can increase the power to detect the combined effects of multiple variants within a gene (25,42). We therefore assessed the significance of the combined Fisher’s *T*-statistic for all common SNPs (MAF ≥ 2%; see Materials and Methods for definition of gene positions) in each annotated *Drosophila* gene. Of the 15,683 genes in the fly genome, 14,146 had at least two SNPs (MAF≥2%). None of the gene-based association tests exceeded the Bonferroni-corrected significance threshold (*p* = 3.53×10^−06^, Figure 3 and Supplementary Figure 9).

The top 30 genes are listed in Supplementary Table 4. In order to allow for SNPs in regulatory regions, we also performed a gene-based analysis with gene positions extended by 5kb from the 5′ and 3′ ends. None of the 15,145 such tests of gene regions with at least two SNPs were significant at a stringent Bonferroni threshold (*p* = 3.30×10^−06^ Supplementary Figures 10, 11; Supplementary Table 5).

Several of the top-ranking genes from the gene-based associations are plausible candidate genes affecting lifespan determination. *CG11523* is predicted to have a *GSK3ß* interaction domain (GSKIP; InterPro term: IPR023231). The human ortholog (GSKIP) protein interacts *in vitro* and *in vivo* with GSK3ß and inhibits the GSK3ß activity (43). Mouse *GSK3ß* is a critical regulator of mTORC1, autophagy, and ageing (44). In addition, *GSK3ß* controls the activity of S6 kinase 1, a crucial component of the TOR pathway, in human cell lines (45). *CG6030* is ranked 10^th^ in our gene-based analysis. *CG6030* encodes the ATP synthase subunit delta, part of the complex V of the mitochondrial electron transport chain, which is the main energy-generating complex (46). Its role in ageing/lifespan is currently unknown, although the inhibition of a different subunit from the same complex (subunit ß) has been shown to extend lifespan in worms (47). *Neprilysin 1* (*Nep1*) was the top-ranked gene-based association when genes were extended to include 5kb from the 5′ and 3′ ends. Thus, it is possible that several SNPs within the regulatory regions of *Nep1* could contribute to determination of female lifespan in these lines. *Nep1* is integral for female fertility (48) and likely to be essential for female reproductive fitness. Fertility has been previously shown to be associated with ageing, that is, the “trade-off” theory of ageing, although the relationship is unclear and the effect of fertility can be separated from lifespan (49). Furthermore, *Nep1* is the major ß-amyloid degrading enzyme in mice and humans, and is associated with progressive Nep1 protein level decline with age in mice (50,51).

Many of the top genes from the gene-based analyses are computationally predicted and have no defined biological processes or molecular functions. The proportion of predicted genes was 36.7% (11 genes) and 53% (16 genes) among the top 30 genes from the strict and extended (genes ± 5kb) gene-based analyses, respectively.

### IIS/TOR Pathway Enrichment

**Figure 4.**
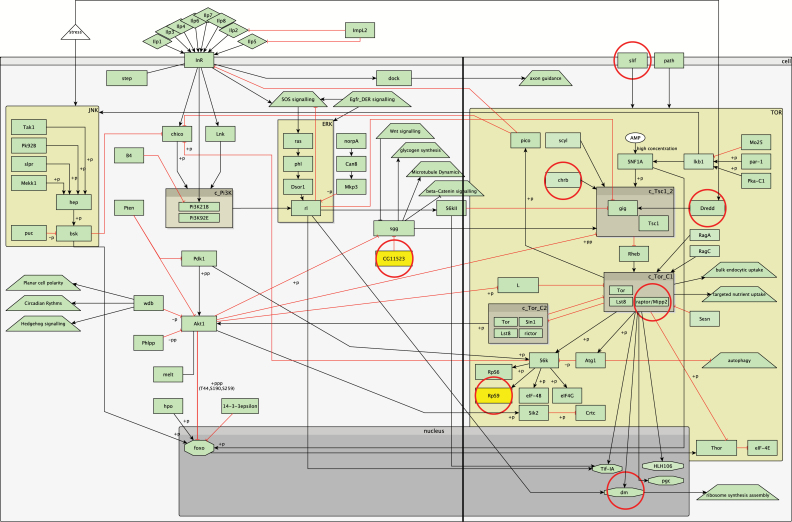
Overview of the insulin/insulin-like growth factor signaling (IIS) and target of rapamycin pathway (TOR) pathways. Rectangles represent genes; diamonds: molecules; triangles: environmental factors; trapezoids: other than IIS or TOR pathways; octagons: transcription factors; arrow lines represent activation; red t-shaped lines represent inhibition; brown boxes starting with c_ represent complexes; yellow boxes represent putative genes part of the IIS (CG11523) or TOR (RpS9) pathways; genes circled in red represent genes found in the top-ranked genes in the gene-based analysis; black horizontal line represents a symbolic separation between the TOR and IIS pathways.

Mutations in several genes in the IIS and TOR pathways affect lifespan (1,3), and there is a considerable literature on their effects and interactions. We previously used this knowledge to build a comprehensive, manually curated signaling network model of the TOR and IIS pathways (2,52). We assessed whether the top-ranked genes in our gene-based analyses were significantly enriched for genes in the IIS and TOR pathways (Figure 4; Supplementary Table 6).

*Chrb*, *slif*, *Mipp2*, *dm*, and *dredd* are in the TOR pathway, and ranked 33^rd^, 36^th^, 38^th^, 71^st^, and 93^rd^, respectively, in the extended gene-based analysis. *Diminutive* is downstream of the TOR pathway (2,52) and heterozygous mutants have an increased lifespan (53). Similarly, *Mipp2* is a crucial component of the TORC1 complex (http://flybase.org (54)), part of the TOR pathway and *slif* is an amino acid transporter that activates TOR signaling in the fat body (55). Further, *charybde* is upstream of the TSC1 complex and inhibits growth, possibly via down-regulating S6K activity (56). *chrb* could also have a protective effect under starvation conditions, as *chrb* mutants are short-lived than controls under DR, and over-expression of *scyl* and *chrb* extends lifespan twofold under DR (56).

The probability of observing five or more genes of 15,145 tested among the top 100 genes is *p* = 4.79×10^−06^ (using a hypergeometric test); therefore, there is a statistically significant enrichment of TOR-related genes within the top-ranked genes. There was no statistically significant enrichment of IIS-related genes within the top 100 genes (data not shown).

*Mipp2* is 4,915bp away from *Nep1*, the top-ranked gene in the extended gene-based analysis. Therefore the signal from the variants in this intergenic region could affect *Mipp2*, *Nep1*, or both genes (Supplementary Figure 12). If we exclude *Mipp2* from the top 100 ranked genes, the probability of observing four or more TOR pathway genes is still significant (*p* = 1.1×10^−04^). Further, *ribosomal protein 9* (*RpS9*), which ranked ninth in the extended gene-based analysis, very likely acts downstream of *S6k*, along with *RpS6* (57–59). Thus, potentially more genes in the top-ranked genes are part of the TOR pathway.

Three genes in the TOR pathway, *chrb*, *dredd*, and *dm*, are ranked 20^th^, 77^th^, and 84^th^, respectively, in the gene-based analyses of variants within genes. The probability of observing three or more genes in the top 100 genes is *p* = 2.42×10^−03^. In addition, *Organic cation transporter 2* (*Orct2*) acts immediately downstream of *S6k* and is required for the control of growth and proliferation (60). In our gene-based analysis *Orct*, another organic cation transporter, was ranked 42^nd^. Both *Orct* and *Orct2* are 73% identical and 84% similar at the amino acid level (Supplementary Figure 13; similar aminoacid residues based on the BLOSUM62 matrix). It is possible that *Orct* could also act downstream of *S6k*. *RpS9* ranked 44^th^ in the strict gene-based analysis, and, as noted above, is likely to be downstream of *S6k*. Thus, a relatively large number of TOR-related genes are found to be significantly enriched in our gene-based analyses.

### Gene Ontology and InterPro Analysis

We performed GO term and InterPro domain enrichment analyses, based on the ranks of the genes from the strict and extended gene-based tests. We did not find significantly enriched/over-represented GO categories (Supplementary Tables 7, 8). These results do not contradict our previous analysis showing enrichment of genes in the TOR pathway. The GO enrichment analysis (using Catmap (28)) tests if genes belonging to a GO category have a higher mean rank compared to the rest of the gene list. Although several genes in the TOR pathway are indeed at the top of the list, there are many others at the bottom of the gene list, thus rendering the overall mean rank of the TOR pathway not statistically significant.

Our InterPro analysis for the extended gene-based tests reveals that several domains are significantly enriched (using a Bonferroni threshold of *p* = 1.66×10^−05^, correcting for all 3011 InterPro terms tested) and potentially associated with lifespan (data not shown). These included DUF227, CHK kinase-like and ALMS motif. The majority of these domains are of unknown function. DUF227 was significantly enriched in *daf-2 C. elegans* mutants (microarray transcriptional profiling (61)). Genes containing DUF227 domain may act downstream of the Insulin receptor *daf-2*, at least in worms. Furthermore, this InterPro term was significantly enriched in flies treated with the xenobiotic phenobarbital (62), suggesting that genes with such a structural domain might play an important role in xenobiotic detoxification and metabolism and have been implicated in insecticide resistance (63).

### Polygenic Score Analysis

**Figure 5.**
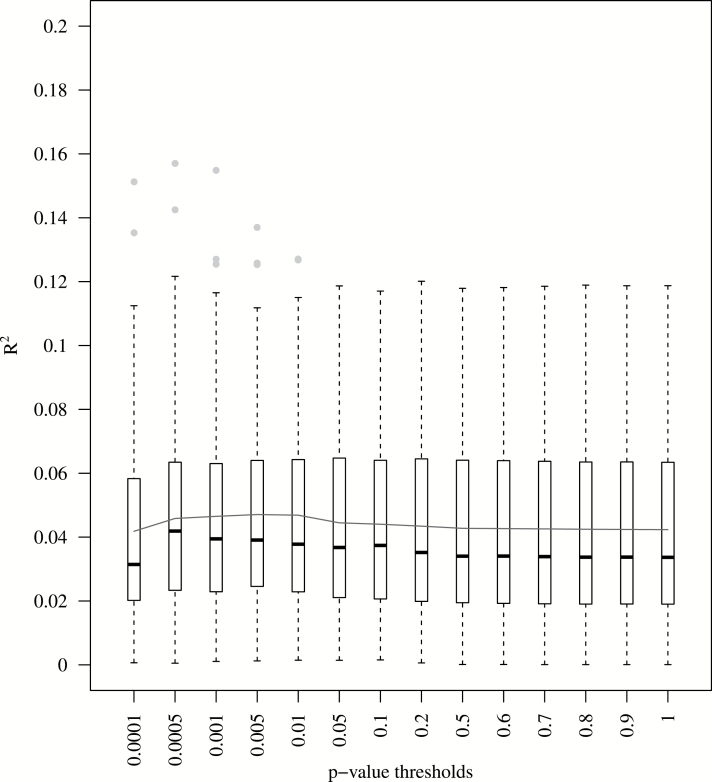
Polygenic score and lifespan. Each box represents the interquartile range (IQR) with the median as a black horizontal line; the whiskers represent values 1.5*IQR; outliers are represented as separate points. The continuous black horizontal line connects the means within each *p* value threshold.

We performed a polygenic score analysis (26) to assess the extent to which the additive effect of common genetic variants predict lifespan. Although the broad-sense heritability of line means is *H* ^2^ = 0.413, we were only able to achieve a maximum mean *R* ^2^ = 0.047±0.0047 (±σ; for SNPs with *p* ≤ 0.005; Figure 5). Thus, only 4.7% of the phenotypic variation in lifespan is explained by the additive effect of common variants.

Estimates of *R* ^2^ using polygenic score analysis can be inflated if the sample size is small (64). We performed 100 permutations using the same procedure for calculating the polygenic scores and *R* ^2^, but randomly permuting the lifespan phenotypes, to estimate *R* ^2^ when there is no association between the phenotype and genotype. The results from these random permutations suggest that lifespan variation could be explained by no more than 1.99% (*p*_*t*_ ≤ 0.0005; *R* ^2^ = 0.019±0.0019), when the phenotype is not associated with the genetic variants (Supplementary Figure 14; Supplementary Table 9). Furthermore, for all *p* value thresholds the mean variation explained was in all cases significantly higher than random permutations of lifespan (*p* ≤ 4×10^−09^ for all *p* value thresholds; Supplementary Table 9). Therefore, the *R* ^2^ estimates from the polygenic score analysis are unlikely to be due to chance. We can conclude that a relatively small proportion (~5%) of variation in lifespan in the DGRP is likely to be due to the additive effect of common genetic variants. The phenotypic variance of lifespan explained by common variants in the polygenic score analysis is similar to that obtained for starvation resistance (median *R* ^2^ = 0.074) and startle response (median *R* ^2^ = 0.08) with genotypic data for the same fly lines (65).

## Discussion

We performed a GWAS of virgin female lifespan using the DGRP (20,21). The primary single-SNP GWAS did not reveal any variants that passed the genome-wide Bonferroni significance threshold. Power calculations suggest that 350–400 lines would be needed to detect single variants with a small to modest effects (<10 days) with > 80% power for MAF < 0.3. This suggests that common alleles (MAF ≥ 2%) with relatively large effects on lifespan are not segregating in the DGRP. Several of the top SNPs are within genes that have been previously shown to impact longevity. The remaining genes tagged by the top SNPs are largely uncharacterized and are novel candidates, likely affecting lifespan determination and ageing.

### Gene-Based Association Analysis

We showed that TOR-related genes are significantly enriched among the top-ranked genes in our gene-based analysis, corroborating that genes shown to be important in lifespan determination and/or ageing are found among the top-ranked genes in our analysis. Thus, the other top-ranked genes are likely to be promising longevity candidates as well. The finding that alleles within TOR-related genes are enriched and found associated with lifespan within these fly lines does not necessary mean that TOR-related genes have the highest effect on lifespan. Since gene mutants within the TOR pathway have been known for some time to have a significant impact on longevity and we have a better understanding of their effect and relationship, we are more likely to detect their effect. This scenario does not negate the significance/importance and potential role of these genes in lifespan determination in the DGRP. Furthermore, a large proportion of the genes ranked at the top of the gene-based analysis are currently without a known function. It is nearly impossible to determine the biological function of these genes or assess their significance with respect to longevity without detailed lab-based experiments. Similar observations, that is, large number of uncharacterized genes, were also made in two separate genome screens (*P*-element insertions (66) and sequencing of flies divergent for late-age fertility and lifespan (67)). Given the highly ranked genes and genes ±5kb with previously known association with lifespan and ageing, as well as the enrichment in TOR-related genes, the highly ranked uncharacterized genes present good candidates for further investigation with respect to lifespan.

We also draw conclusions based on the gene-based analysis of SNPs within genes and genes ± 5kb almost interchangeably. Without additional transcriptome-based data and additional analysis (eg, eQTLs) we are unable to determine if SNPs in gene regulatory regions (eg, promoter regions) are likely or not to affect transcription of these genes and subsequently affect lifespan. Additional transcriptome-based data or experimental assays could shed light on the effect of such regulatory elements on the expression of such genes and their effect on lifespan.

### Gene Ontology and InterPro Analysis

Around half of the top 20 GO categories in the gene-based analysis are related to carbohydrate metabolism. This certainly implies that genes up or downstream of nutrient-sensing pathways, such as IIS or TOR, are likely to play a crucial role in lifespan determination in the DGRP. Apart from several statistically enriched InterPro terms, the rest of the top InterPro/GO terms and associated genes have thus far not been shown to be involved in lifespan determination. These could potentially be involved in biological mechanisms of ageing that are novel and could shed some light on biological pathways previously not implied in ageing. In addition, a large proportion of the top genes in our gene-based analysis are currently without a clearly defined function, hence we cannot utilize these in any meaningful way. Clearly, the GO and InterPro terms rely on previously defined biological and molecular function of such genes, thus these genes will be missed in any high-level analysis.

### Genetic Contribution to Lifespan

We also explored how much of the phenotypic variance of lifespan, is potentially explicable in terms of common genetic variants. We showed that common genetic variants significantly contribute to lifespan determination, as compared to a randomly permuted lifespan data, whereby the relationship between the genotypes and phenotype is interrupted. Nevertheless, the additive effects of common genetic variants only explain ~5% of the phenotypic variance, in agreement with similar analyses of other phenotypes in the DGRP (65). The low phenotypic variance explained could be due to an increased variability in the lifespan determination of each individual line and of course all pairwise and higher order epistatic interaction, which are not interrogated using the additive, infinitesimal model.

Rare genetic variants are also likely to play a significant role in lifespan determination within these fly lines. These variants were excluded from analysis in this study, due to the minimal power to detect an association with such variants.

## Conclusions

We utilized a multifaceted, hypothesis-free, and unbiased genome-wide approach in order to identify genes and pathways are associated with variation in lifespan. The analysis was based on whole-genome sequencing data, part of the valuable community resource: DGRP. Even though our results suggest that there is little power to detect single-SNP associations with small effects on lifespan, a gene-based analysis revealed additional information about genes and pathways that are likely to have an impact on lifespan, including several genes in the TOR pathway that have been previously shown to have an impact on lifespan. Experiments are now needed to validate or refute the observed associations.

## Supplementary Material

Supplementary material can be found at: http://biomedgerontology.oxfordjournals.org/

## Funding

D.K.I. was funded by the Wellcome Trust Strategic Award at UCL (081394/B/06/Z and 098565/Z/12/Z). This research was partially supported by NIH grants (R01 GM45246 and R01 AG043490 to T.F.C.M.).

## Supplementary Material

Supplementary Data

## References

[1] Kapahi P, Zid BM, Harper T, Koslover D, Sapin V, Benzer S. Regulation of lifespan in Drosophila by modulation of genes in the TOR signaling pathway. Curr Biol. 2004;14:885–890, doi:10.1016/j.cub.2004.03.059. 10.1016/j.cub.2004.03.059 PMC2754830 15186745

[2] Ivanov DK, Papatheodorou I, Ziehm M, Thornton JM. Transcriptional feedback in the insulin signalling pathway modulates ageing in both Caenorhabditis elegans and Drosophila melanogaster. Mol Biosyst. 2013;9:1756–1764, doi:10.1039/c3mb25485b. 10.1039/c3mb25485b PMC3693544 23624434

[3] Fontana L Partridge L Longo VD Extending healthy life span–from yeast to humans. Science. 2010;328:321–326, doi:10.1126/science.1172539. 20395504 10.1126/science.1172539 PMC3607354

[4] Kenyon CJ The genetics of ageing. Nature. 2010;464:504–512, doi:10.1038/nature08980. 20336132 10.1038/nature08980

[5] Zhang Y Bokov A Gelfond J Rapamycin extends life and health in C57BL/6 mice. J Gerontol A Biol Sci Med Sci. 2014;69:119–130, doi:10.1093/gerona/glt056. 23682161 10.1093/gerona/glt056 PMC4038246

[6] Murabito JM Yuan R Lunetta KL The search for longevity and healthy aging genes: insights from epidemiological studies and samples of long-lived individuals. J Gerontol A Biol Sci Med Sci. 2012;67:470–479, doi:10.1093/gerona/gls089. 22499766 10.1093/gerona/gls089 PMC3326242

[7] Hjelmborg vBJ Iachine I Skytthe A Genetic influence on human lifespan and longevity. Hum Genet. 2006;119:312–321, doi:10.1007/s00439-006-0144-y. 16463022 10.1007/s00439-006-0144-y

[8] McGue M Vaupel JW Holm N Harvald B Longevity is moderately heritable in a sample of Danish twins born 1870-1880. J Gerontol. 1993;48:B237–B244, doi:10.1093/geronj/48.6.B237. 8227991 10.1093/geronj/48.6.b237

[9] Deelen J Beekman M Uh HW Genome-wide association study identifies a single major locus contributing to survival into old age; the APOE locus revisited. Aging Cell. 2011;10:686–698, doi:10.1111/j.1474-9726.2011.00705.x. 21418511 10.1111/j.1474-9726.2011.00705.x PMC3193372

[10] Nebel A Kleindorp R Caliebe A A genome-wide association study confirms APOE as the major gene influencing survival in long-lived individuals. Mech Ageing Dev. 2011;132:324–330, doi:10.1016/j.mad.2011.06.008. 21740922 10.1016/j.mad.2011.06.008

[11] Broer L Buchman AS Deelen J GWAS of longevity in CHARGE consortium confirms APOE and FOXO3 candidacy. J Gerontol A Biol Sci Med Sci. 2015;70:110–118, doi:10.1093/gerona/glu166. 25199915 10.1093/gerona/glu166 PMC4296168

[12] Jacobsen R Martinussen T Christiansen L Increased effect of the ApoE gene on survival at advanced age in healthy and long-lived Danes: two nationwide cohort studies. Aging Cell. 2010;9:1004–1009, doi:10.1111/j.1474-9726.2010.00626.x. 20849521 10.1111/j.1474-9726.2010.00626.x PMC2988163

[13] Morris BJ Donlon TA He Q Genetic analysis of TOR complex gene variation with human longevity: a nested case-control study of American men of Japanese ancestry. J Gerontol A Biol Sci Med Sci. 2015;70:133–142, doi:10.1093/gerona/glu021. 24589862 10.1093/gerona/glu021 PMC4366598

[14] Deelen J Uh HW Monajemi R Gene set analysis of GWAS data for human longevity highlights the relevance of the insulin/IGF-1 signaling and telomere maintenance pathways. Age (Dordr). 2013;35:235–249, doi:10.1007/s11357-011-9340-3. 22113349 10.1007/s11357-011-9340-3 PMC3543749

[15] Flachsbart F Caliebe A Kleindorp R Association of FOXO3A variation with human longevity confirmed in German centenarians. Proc Natl Acad Sci USA. 2009;106:2700–2705, doi:10.1073/pnas.0809594106. 19196970 10.1073/pnas.0809594106 PMC2650329

[16] Soerensen M Dato S Christensen K Replication of an association of variation in the FOXO3A gene with human longevity using both case-control and longitudinal data. Aging Cell. 2010;9:1010–1017, doi:10.1111/j.1474-9726.2010.00627.x. 20849522 10.1111/j.1474-9726.2010.00627.x PMC2992870

[17] Kahn AJ FOXO3 and related transcription factors in development, aging, and exceptional longevity. J Gerontol A Biol Sci Med Sci. 2015;70:421–425, doi:10.1093/gerona/glu044. 24747665 10.1093/gerona/glu044 PMC4447793

[18] Walter S Atzmon G Demerath EW Garcia ME Kaplan RC Kumari M A genome-wide association study of aging. Neurobiol Aging. 2011;32:2109, doi:10.1016/j.neurobiolaging.2011.05.026. 21782286 10.1016/j.neurobiolaging.2011.05.026 PMC3193030

[19] Minster RL Sanders JL Singh J Genome-wide association study and linkage analysis of the healthy aging index. J Gerontol A Biol Sci Med Sci. 2015, doi:10.1093/gerona/glv006. 10.1093/gerona/glv006 PMC4506316 25758594

[20] Mackay TF Richards S Stone EA The Drosophila melanogaster Genetic Reference Panel. Nature. 2012;482:173–178, doi:10.1038/nature10811. 22318601 10.1038/nature10811 PMC3683990

[21] Huang W Massouras A Inoue Y Peiffer J Ramia M Tarone A Natural variation in genome architecture among 205 *Drosophila melanogaster* Genetic Reference Panel lines. Genome Res. 2014;24:1193–1208, doi:10.1101/gr.171546.113. 10.1101/gr.171546.113 PMC4079974 24714809

[22] Arya GH Weber AL Wang P Natural variation, functional pleiotropy and transcriptional contexts of odorant binding protein genes in Drosophila melanogaster. Genetics. 2010;186:1475–1485, doi:10.1534/genetics.110.123166. 20870963 10.1534/genetics.110.123166 PMC2998325

[23] Price AL Patterson NJ Plenge RM Weinblatt ME Shadick NA Reich D Principal components analysis corrects for stratification in genome-wide association studies. Nat Genet. 2006;38:904–909, doi:10.1038/ng1847. 16862161 10.1038/ng1847

[24] Purcell S Neale B Todd-Brown K PLINK: a tool set for whole-genome association and population-based linkage analyses. Am J Hum Genet. 2007;81:559–575, doi:10.1086/519795. 17701901 10.1086/519795 PMC1950838

[25] Moskvina V Craddock N Holmans P Gene-wide analyses of genome-wide association data sets: evidence for multiple common risk alleles for schizophrenia and bipolar disorder and for overlap in genetic risk. Mol Psychiatry. 2009;14:252–260, doi:10.1038/mp.2008.133. 19065143 10.1038/mp.2008.133 PMC3970088

[26] Purcell SM, Wray NR, Stone JL, et al. Common polygenic variation contributes to risk of schizophrenia and bipolar disorder. Nature. 2009;460:748–752, doi:10.1038/nature08185. 10.1038/nature08185 PMC3912837 19571811

[27] Hunter S Jones P Mitchell A InterPro in 2011: new developments in the family and domain prediction database. Nucleic Acids Res. 2012;40:D306–312, doi:10.1093/nar/gkr948. 22096229 10.1093/nar/gkr948 PMC3245097

[28] Breslin T Edén P Krogh M Comparing functional annotation analyses with Catmap. BMC Bioinformatics. 2004;5:193, doi:10.1186/1471-2105-5–193. 15588298 10.1186/1471-2105-5-193 PMC543458

[29] Mockett RJ Cockrell JC Puri S Nguyen M Nisa M Long-lived genotypes for studies of life extension in Drosophila melanogaster. Mech Ageing Dev. 2012;133:359–367, doi:10.1016/j.mad.2012.04.002. 22525703 10.1016/j.mad.2012.04.002

[30] Grönke S Clarke DF Broughton S Andrews TD Partridge L Molecular evolution and functional characterization of Drosophila insulin-like peptides. PLoS Genet. 2010;6:e1000857, doi:10.1371/journal.pgen.1000857. 20195512 10.1371/journal.pgen.1000857 PMC2829060

[31] Ziehm M Piper MD Thornton JM Analysing variation in Drosophila aging across independent experimental studies: a meta-analysis of survival data. Aging Cell. 2013;12:917–922, doi:10.1111/acel.12123. 23795998 10.1111/acel.12123 PMC3963443

[32] Zid BM Rogers AN Katewa SD 4E-BP extends lifespan upon dietary restriction by enhancing mitochondrial activity in Drosophila. Cell. 2009;139:149–160, doi:10.1016/j.cell.2009.07.034. 19804760 10.1016/j.cell.2009.07.034 PMC2759400

[33] Partridge L Alic N Bjedov I Piper MD Ageing in Drosophila: the role of the insulin/Igf and TOR signalling network. Exp Gerontol. 2011;46:376–381, doi:10.1016/j.exger.2010.09.003. 20849947 10.1016/j.exger.2010.09.003 PMC3087113

[34] Grönke S Mildner A Fellert S Brummer lipase is an evolutionary conserved fat storage regulator in Drosophila. Cell Metab. 2005;1:323–330, doi:10.1016/j.cmet.2005.04.003. 16054079 10.1016/j.cmet.2005.04.003

[35] Rogina B Helfand SL Frankel S Longevity regulation by Drosophila Rpd3 deacetylase and caloric restriction. Science. 2002;298:1745, doi:10.1126/science.1078986. 12459580 10.1126/science.1078986

[36] Zhou D Xue J Lai JC Schork NJ White KP Haddad GG Mechanisms underlying hypoxia tolerance in Drosophila melanogaster: hairy as a metabolic switch. PLoS Genet. 2008;4:e1000221, doi:10.1371/journal.pgen.1000221. 18927626 10.1371/journal.pgen.1000221 PMC2556400

[37] Irisarri M Lavista-Llanos S Romero NM Centanin L Dekanty A Wappner P Central role of the oxygen-dependent degradation domain of Drosophila HIFalpha/Sima in oxygen-dependent nuclear export. Mol Biol Cell. 2009;20:3878–3887, doi:10.1091/mbc.E09-01-0038. 19587118 10.1091/mbc.E09-01-0038 PMC2735486

[38] Mabon ME Scott BA Crowder CM Divergent mechanisms controlling hypoxic sensitivity and lifespan by the DAF-2/insulin/IGF-receptor pathway. PLoS One. 2009;4:e7937, doi:10.1371/journal.pone.0007937. 19936206 10.1371/journal.pone.0007937 PMC2775958

[39] Zhang L Ebenezer PJ Dasuri K Aging is associated with hypoxia and oxidative stress in adipose tissue: implications for adipose function. Am J Physiol Endocrinol Metab. 2011;301:E599–E607, doi:10.1152/ajpendo.00059.2011. 21586698 10.1152/ajpendo.00059.2011 PMC3275102

[40] Leiser SF Begun A Kaeberlein M HIF-1 modulates longevity and healthspan in a temperature-dependent manner. Aging Cell. 2011;10:318–326, doi:10.1111/j.1474–9726.2011.00672.x. 21241450 10.1111/j.1474-9726.2011.00672.x PMC3980873

[41] Feng J Bussière F Hekimi S Mitochondrial electron transport is a key determinant of life span in Caenorhabditis elegans. Dev Cell. 2001;1:633–644, doi:10.1016/S1534-5807(01)00071-5. 11709184 10.1016/s1534-5807(01)00071-5

[42] Neale BM Sham PC The future of association studies: gene-based analysis and replication. Am J Hum Genet. 2004;75:353–362, doi:10.1086/423901. 15272419 10.1086/423901 PMC1182015

[43] Chou HY Howng SL Cheng TS GSKIP is homologous to the Axin GSK3beta interaction domain and functions as a negative regulator of GSK3beta. Biochemistry. 2006;45:11379–11389, doi:10.1021/bi061147r. 16981698 10.1021/bi061147r

[44] Zhou J Freeman TA Ahmad F Shang X Mangano E Gao E *GSK-3alpha* is a central regulator of age-related pathologies in mice. J Clin Invest. 2013;123:1821–1832, doi:10.1172/JCI64398. 23549082 10.1172/JCI64398 PMC3613907

[45] Shin S Wolgamott L Yu Y Blenis J Yoon SO Glycogen synthase kinase (GSK)-3 promotes p70 ribosomal protein S6 kinase (p70S6K) activity and cell proliferation. Proc Natl Acad Sci USA. 2011;108:E1204–E1213, doi:10.1073/pnas.1110195108. 22065737 10.1073/pnas.1110195108 PMC3223461

[46] Boyer PD The ATP synthase–a splendid molecular machine. Annu Rev Biochem. 1997;66:717–749, doi:10.1146/annurev.biochem.66.1.717. 9242922 10.1146/annurev.biochem.66.1.717

[47] Chin RM Fu X Pai MY Vergnes L Hwang H Deng G The metabolite alpha-ketoglutarate extends lifespan by inhibiting ATP synthase and TOR. Nature. 2014;510:397–401, doi:10.1038/nature13264. 24828042 10.1038/nature13264 PMC4263271

[48] Sitnik JL Francis C Hens K Huybrechts R Wolfner MF Callaerts P Neprilysins: an evolutionarily conserved family of metalloproteases that play important roles in reproduction in Drosophila. Genetics. 2014;196:781–797, doi:10.1534/genetics.113.160945. 24395329 10.1534/genetics.113.160945 PMC3948806

[49] Flatt T Survival costs of reproduction in Drosophila. Exp Gerontol. 2011;46:369–375, doi:10.1016/j.exger.2010.10.008. 20970491 10.1016/j.exger.2010.10.008

[50] Apelt J Ach K Schliebs R Aging-related down-regulation of neprilysin, a putative beta-amyloid-degrading enzyme, in transgenic Tg2576 Alzheimer-like mouse brain is accompanied by an astroglial upregulation in the vicinity of beta-amyloid plaques. Neurosci Lett. 2003;339:183–186, doi:10.1016/S0304–3940(03)00030-2. 12633883 10.1016/s0304-3940(03)00030-2

[51] Hellström-Lindahl E Ravid R Nordberg A Age-dependent decline of neprilysin in Alzheimer’s disease and normal brain: inverse correlation with A beta levels. Neurobiol Aging. 2008;29:210–221, doi:10.1016/j.neurobiolaging.2006.10.010. 17098332 10.1016/j.neurobiolaging.2006.10.010

[52] Papatheodorou I Ziehm M Wieser D Alic N Partridge L Thornton JM Using answer set programming to integrate RNA expression with signalling pathway information to infer how mutations affect ageing. PLoS One. 2012;7:e50881, doi:10.1371/journal.pone.0050881. 23251396 10.1371/journal.pone.0050881 PMC3519537

[53] Greer C Lee M Westerhof M Myc-dependent genome instability and lifespan in Drosophila. PLoS One. 2013;8:e74641, doi:10.1371/journal.pone.0074641. 24040302 10.1371/journal.pone.0074641 PMC3765364

[54] St Pierre SE Ponting L Stefancsik R McQuilton P FlyBase 102--advanced approaches to interrogating FlyBase. Nucleic Acids Res. 2014;42:D780–D788, doi:10.1093/nar/gkt1092. 24234449 10.1093/nar/gkt1092 PMC3964969

[55] Colombani J Raisin S Pantalacci S Radimerski T Montagne J Léopold P A nutrient sensor mechanism controls Drosophila growth. Cell. 2003;114:739–749, doi:10.1016/S0092–8674(03)00713-X. 14505573 10.1016/s0092-8674(03)00713-x

[56] Reiling JH Hafen E The hypoxia-induced paralogs Scylla and Charybdis inhibit growth by down-regulating S6K activity upstream of TSC in Drosophila. Genes Dev. 2004;18:2879–2892, doi:10.1101/gad.322704. 15545626 10.1101/gad.322704 PMC534649

[57] O’Donohue MF Choesmel V Faubladier M Fichant G Gleizes PE Functional dichotomy of ribosomal proteins during the synthesis of mammalian 40S ribosomal subunits. J Cell Biol. 2010;190:853–866, doi:10.1083/jcb.201005117. 20819938 10.1083/jcb.201005117 PMC2935573

[58] Liu S Lu B Reduction of protein translation and activation of autophagy protect against PINK1 pathogenesis in Drosophila melanogaster. PLoS Genet. 2010;6:e1001237, doi:10.1371/journal.pgen.1001237. 21151574 10.1371/journal.pgen.1001237 PMC3000346

[59] Lindström MS Nistér M Silencing of ribosomal protein S9 elicits a multitude of cellular responses inhibiting the growth of cancer cells subsequent to p53 activation. PLoS One. 2010;5:e9578, doi:10.1371/journal.pone.0009578. 20221446 10.1371/journal.pone.0009578 PMC2833189

[60] Herranz H Morata G Milán M calderón encodes an organic cation transporter of the major facilitator superfamily required for cell growth and proliferation of Drosophila tissues. Development. 2006;133:2617–2625, doi:10.1242/dev.02436. 16774996 10.1242/dev.02436

[61] McElwee JJ Schuster E Blanc E Thomas JH Gems D Shared transcriptional signature in Caenorhabditis elegans Dauer larvae and long-lived daf-2 mutants implicates detoxification system in longevity assurance. J Biol Chem. 2004;279:44533–44543, doi:10.1074/jbc.M406207200. 15308663 10.1074/jbc.M406207200

[62] King-Jones K Horner MA Lam G Thummel CS The DHR96 nuclear receptor regulates xenobiotic responses in Drosophila. Cell Metab. 2006;4:37–48, doi:10.1016/j.cmet.2006.06.006. 16814731 10.1016/j.cmet.2006.06.006

[63] Aminetzach YT Macpherson JM Petrov DA Pesticide resistance via transposition-mediated adaptive gene truncation in Drosophila. Science. 2005;309:764–767, doi:10.1126/science.1112699. 16051794 10.1126/science.1112699

[64] Wray NR Yang J Hayes BJ Price AL Goddard ME Visscher PM Pitfalls of predicting complex traits from SNPs. Nat Rev Genet. 2013;14:507–515, doi:10.1038/nrg3457. 23774735 10.1038/nrg3457 PMC4096801

[65] Ober U Ayroles JF Stone EA Using whole-genome sequence data to predict quantitative trait phenotypes in Drosophila melanogaster. PLoS Genet. 2012;8:e1002685, doi:10.1371/journal.pgen.1002685. 22570636 10.1371/journal.pgen.1002685 PMC3342952

[66] Magwire MM Yamamoto A Carbone MA Quantitative and molecular genetic analyses of mutations increasing Drosophila life span. PLoS Genet. 2010;6:e1001037, doi:10.1371/journal.pgen.1001037. 20686706 10.1371/journal.pgen.1001037 PMC2912381

[67] Remolina SC Chang PL Leips J Nuzhdin SV Hughes KA Genomic basis of aging and life-history evolution in Drosophila melanogaster. Evolution. 2012;66:3390–3403, doi:10.1111/j.1558-5646.2012.01710.x. 23106705 10.1111/j.1558-5646.2012.01710.x PMC4539122

