# Supplementary material for: Longevity GWAS Using the Drosophila Genetic Reference Panel
Source: J Gerontol A Biol Sci Med Sci. 2015 Apr 28;70(12):1470–8. doi: 10.1093/gerona/glv047 (PMC4631106; doi:10.1093/gerona/glv047)
Supplement: Supplementary Data [file supp_70_12_1470__index.html]

Longevity GWAS Using the Drosophila Genetic Reference Panel — Longevity GWAS Using the Drosophila Genetic Reference Panel — Supplementary Data 

# Longevity GWAS Using the *Drosophila* Genetic Reference Panel

## Supplementary Data

Data files

**Files in this Data Supplement:**

- Supplementary Data - Supplementary Data
