# Supplementary material for: Longevity GWAS Using the Drosophila Genetic Reference Panel
Source: J Gerontol A Biol Sci Med Sci. 2015 Apr 28;70(12):1470–8. doi: 10.1093/gerona/glv047 (PMC4631106; doi:10.1093/gerona/glv047)
Supplement: Supplementary Data [file supp_glv047_Longevity_GWAS_using_DGRP_Ivanov_et_al_Supplementary_Materials.docx]

SupportingInformation

1. Power calculations

For power calculations we used the pwr.t2n.test package in R. Cohen’s effect size *d* was calculated as follows:

$d=\frac{\mu_{1}-\mu_{2}}{\sigma_{p}}$, where $\sigma_{p}=\sqrt{\frac{(n_{1}-1)\sigma_{1}^{2}+(n_{2}-1)\sigma_{2}^{2}}{n_{1}+n_{2}-2}}$; *μ_1_*and *μ_2_* are the mean lifespans associated with each genotype; *σ_p_* is the pooled standard deviation; $\sigma_{1}^{2}$ and $\sigma_{2}^{2}$ are variances associated with the mean lifespans within each genotype. These mean lifespans were taken from the most associated SNP (*2R*_1632386; *p*-value=5.9x10^-08^; *μ_1_*=56.57and *μ_2_*=45.97; $\sigma_{1}^{2}$=89.86 and $\sigma_{2}^{2}$=63.57).

1. Broad sense heritability

To estimate broad sense heritability (*H^2^*) we partitioned the phenotypic variance between lines and the error variance for each line using ANOVA. Broad sense heritability was estimated by $H^{2}=\frac{\sigma_{L}^{2}}{\sigma_{L}^{2}+\sigma_{E}^{2}}$, where $\sigma_{L}^{2}$ is the among-line variance and $\sigma_{E}^{2}$ is the within-line variance.

1. *Drosophila melanogaster*lines

The Drosophila Genetic Reference Panel, Freeze 2.0 ([1](#_ENREF_1), [2](#_ENREF_2)), comprises 205 *D*. *melanogaster*lines derived by 20 generations of full-sib mating from wild-type caught females from Raleigh, North Carolina. Longevity was assayed as previously described ([3](#_ENREF_3), [4](#_ENREF_4)). All flies were reared from egg to adult on 10 ml standard cornmealagar-molasses medium at 25**°** in shell vials. The density of thestocks was controlled for three generations prior to the startof the longevity assays by restricting egg laying to 3 days andinitiating the cultures with 10 pairs of flies. A total of 25 virgin males and females per line were collected in a 24-hr period and 2-day-old flies were housed in five replicate vials with five same-sex individuals per vial. Flies were transferred to fresh medium every 2 days and the number of live flies was recorded until all were dead. The assays were performed in three temporally overlapping blocks; no block effects were observed.**Supplementary Graphs and Tables**

Supplementary Figure 1. SNP call rate. The red horizontal line represents the 0.9 SNP call rate. Coverage represents the proportion of genotypes present per SNP


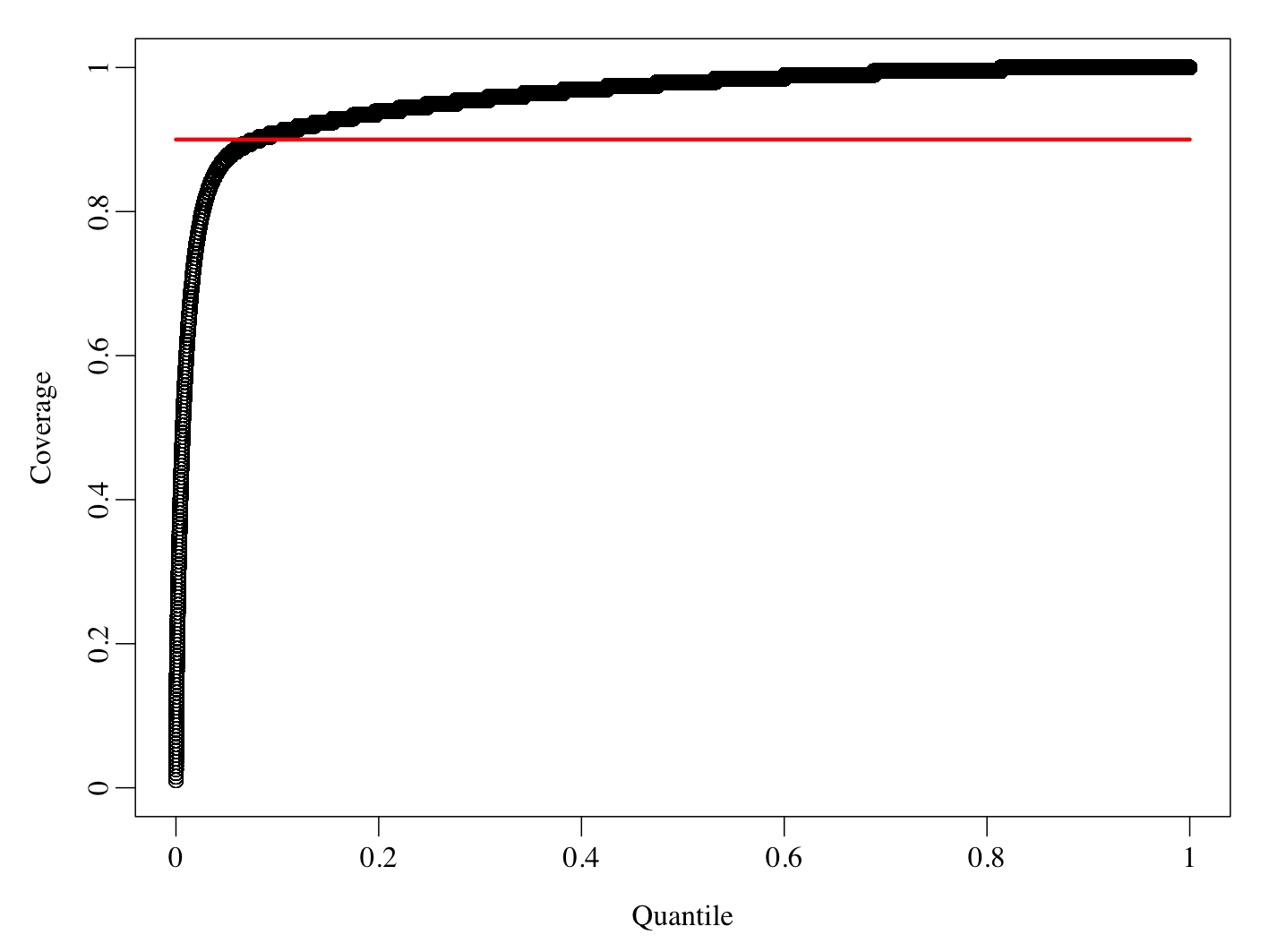


Supplementary Figure 2. Minor allele frequency (MAF). The red verticalline represents MAF=0.02

Supplementary Figure 3. Histogram of individual call rate

Supplementary Figure 4. Individual call rate. Individual call rate represents the proportion of genotypes present per fly

Supplementary Figure 5. Lifespan variation within individual fly lines (165 lines). The red vertical line represents the mean of the standard deviation between the fly lines (10.6)

Supplementary Figure 6. Principal component analysis (PCA), 197 lines. PC1- principal component 1; PC2- principal component 2; PC3- principal component 3; Colours represent the inversion haplotype for *In(3R)Mo* and *In(2L)t*. 00- black, 01- red, 11- light blue, 12- pink, 22- grey, 10- blue, 20- yellow, 02- green


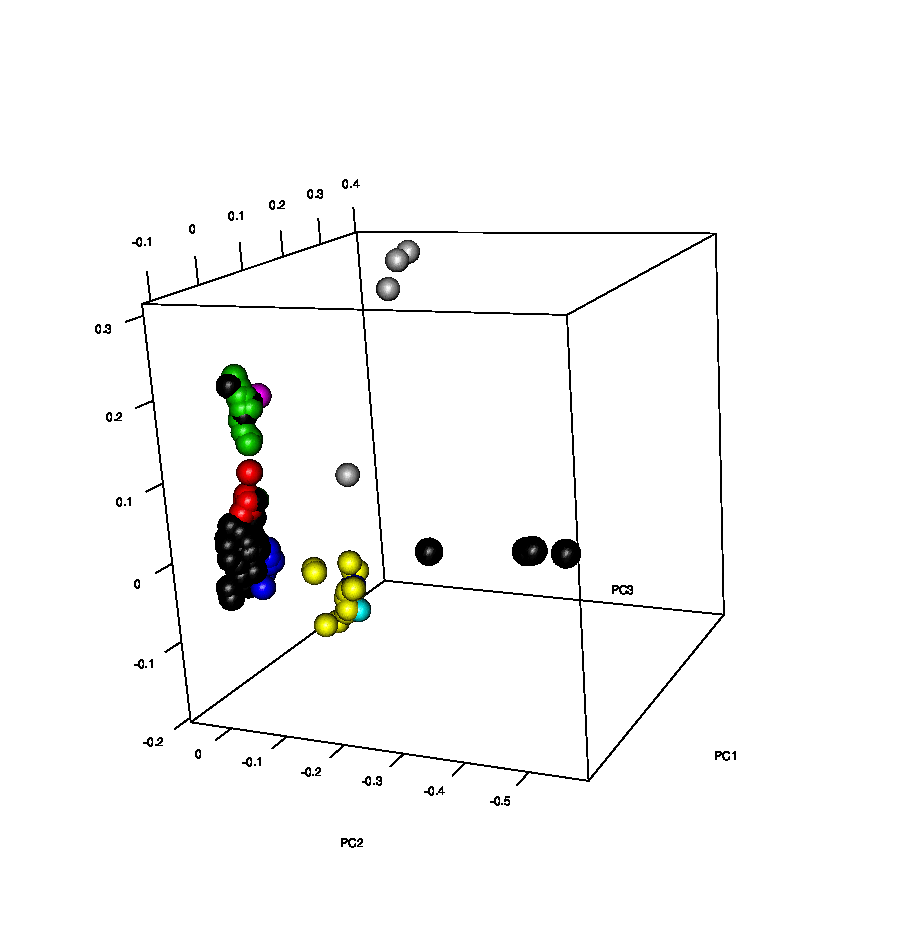


Supplementary Figure 7. SNP-based QQ-plot


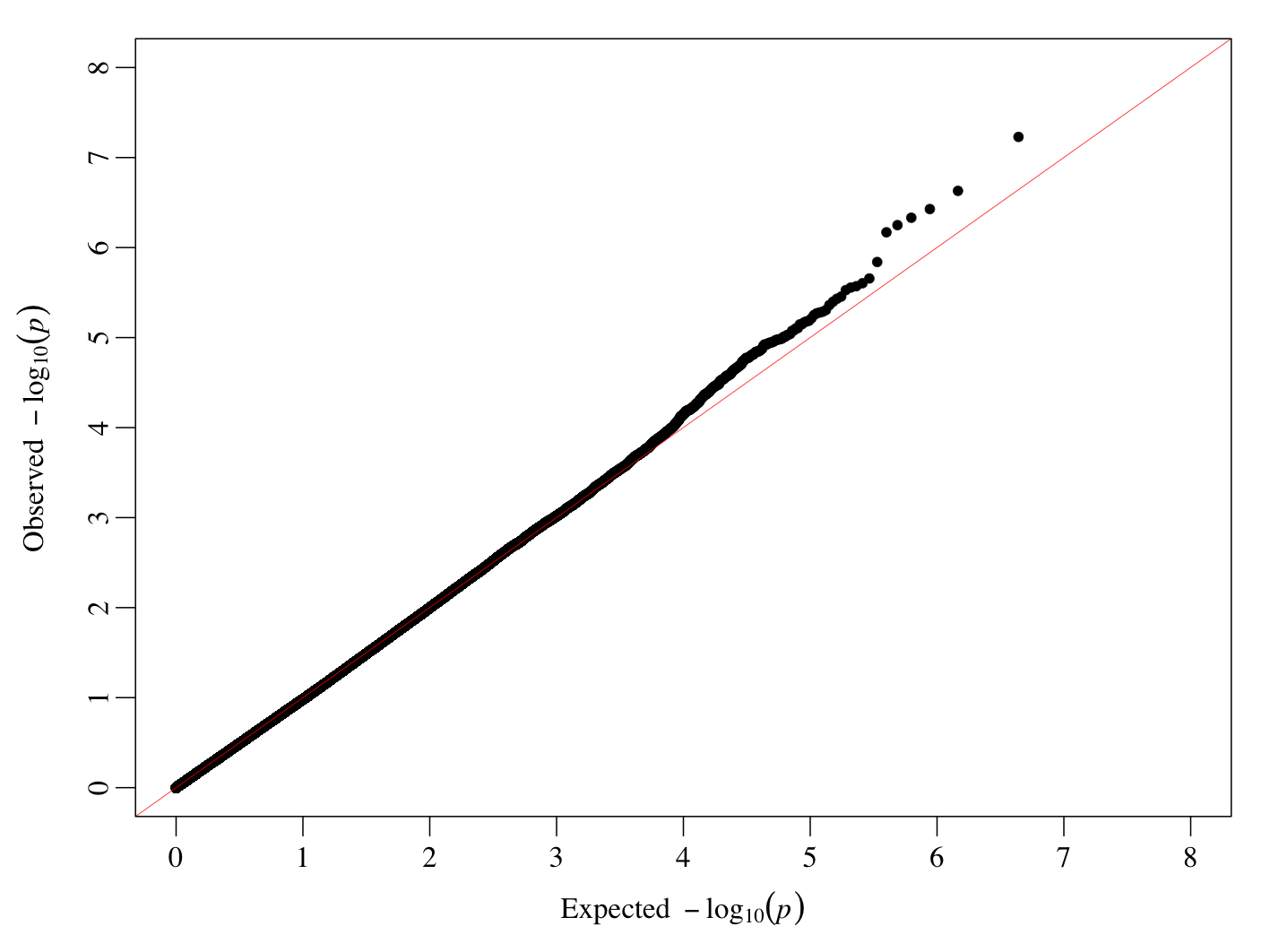


Supplementary Figure 8. Power to detect single-SNP association. Effect size = 10days (Cohen’s *d*=1.15), pooled standard deviation = 9.25, *µ_1_*=56.57, *µ_2_*=45.97; red line represent MAF 0.1, brown line 0.2, green line 0.3, blue line 0.4 and pink line 0.5. The red vertical line represents the DGRP sample size (197 lines)

Supplementary Figure 9. Gene-based QQ-plot

Supplementary Figure 10. Manhattan plot for gene-based analysis (genes ±5kb). Each point represents a gene. The height of the points represents the strength of association with lifespan, expressed as -*log*_10_(*p*-value).The red horizontal line represents genome-wide Bonferroni significance threshold *p*=3.30x10^-06^

Supplementary Figure 11. Gene-based QQ-plot (genes±5kb)

Supplementary Figure 12. LD structure of *Mipp2* and *Nep1*. Each point in the top half of the graph represents a SNP. The y-axis represents the strength of association expressed in –*log*_10_(*p*-value). Red dots represent SNPs with negative *ß* coefficients and green dots SNPs with positive *ß* coefficients. The middle panel shows the positions of *Mipp2* and *Nep1* in the *Drosophila* genome using the UCSC genome browser <http://genome-euro.ucsc.edu/cgi-bin/hgGateway>; The bottom paneldepicts the LD structure within the two genes, expressed in terms of *R^2^*. Black squares represent *R^2^*=1. The LD structure was produced using Haploview ([5](#_ENREF_5))


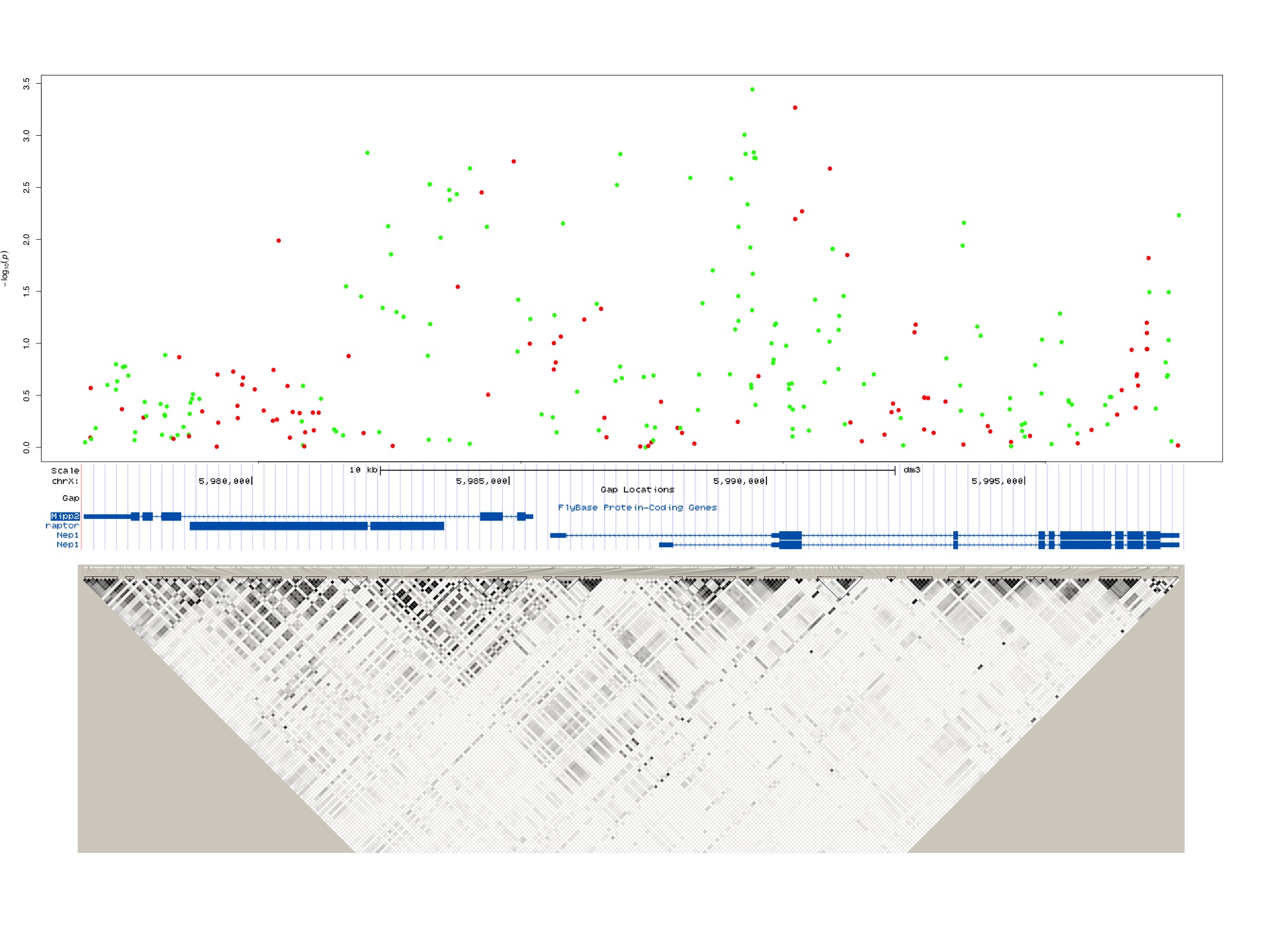


Supplementary Figure 13. Protein alignment of *Orct* (*FBgn0019952*) and *Orct2* (*FBgn0086365*). The protein alignment was produced using ClustalX ([6](#_ENREF_6))

Supplementary Figure 14. Polygenic score (permuted lifespan phenotype). The lifespan data were permuted 100 times. Each box represents the interquartile range (IQR) with the median as a black horizontal line; the whiskers represent values 1.5*IQR; outliers are represented as light blue points. The orange line connects the means within each *p*-value threshold

Supplementary Table 1. DGRP lines, genotypes and phenotypes. ^1^*Wolbachia* status: 1- absent, 2- present;^2^Number of flies used for determining the mean and median lifespan; The lifespan data for several lines were derived from ([3](#_ENREF_3)), where only the mean lifespan was given, hence for standard deviation, number of flies used and median lifespan is given as NA or not assigned.

| Line | *Wolbachia* status^1^ | Mean lifespan | Standard deviation | Number flies used^2^ | Median lifespan | Number missing SNPs | Frequency missing SNPs |
| --- | --- | --- | --- | --- | --- | --- | --- |
| line_21 | 2 | 49.76 | 10.04 | 25 | 51 | 28607 | 0.0130 |
| line_26 | 1 | 46.39 | 9.13 | 23 | 46 | 37755 | 0.0172 |
| line_28 | 1 | 54.00 | 13.13 | 24 | 56 | 23626 | 0.0108 |
| line_31 | 1 | 71.88 | 7.98 | 25 | 71 | 264022 | 0.1204 |
| line_32 | 1 | 52.56 | 6.60 | 25 | 54 | 42210 | 0.0192 |
| line_38 | 1 | 48.27 | 19.75 | 22 | 51 | 131573 | 0.0600 |
| line_40 | 2 | 56.84 | 9.86 | 25 | 58 | 6940 | 0.0032 |
| line_41 | 1 | 59.24 | 23.78 | 25 | 68 | 54027 | 0.0246 |
| line_42 | 1 | 45.80 | 4.26 | 25 | 46 | 35373 | 0.0161 |
| line_45 | 1 | 48.27 | 6.76 | 22 | 47 | 55160 | 0.0251 |
| line_48 | 2 | 57.83 | 13.74 | 24 | 61 | 167964 | 0.0766 |
| line_49 | 2 | 53.84 | 6.11 | 25 | 53 | 177048 | 0.0807 |
| line_57 | 1 | 59.67 | 11.81 | 24 | 60 | 34166 | 0.0156 |
| line_59 | 1 | 58.52 | 22.65 | 25 | 67 | 88646 | 0.0404 |
| line_69 | 2 | 45.13 | 11.41 | 23 | 45 | 82957 | 0.0378 |
| line_73 | 2 | 52.33 | 6.04 | 24 | 51 | 86777 | 0.0396 |
| line_75 | 2 | 44.56 | 15.63 | 25 | 48 | 38276 | 0.0175 |
| line_83 | 1 | 68.40 | 14.24 | 20 | 70 | 33671 | 0.0154 |
| line_85 | 1 | 41.95 | 12.38 | 19 | 44 | 201093 | 0.0917 |
| line_88 | 1 | 58.79 | 11.56 | 24 | 61 | 182007 | 0.0830 |
| line_91 | 1 | 53.64 | 9.08 | 25 | 53 | 71934 | 0.0328 |
| line_93 | 1 | 46.58 | 9.61 | 24 | 50 | 30236 | 0.0138 |
| line_100 | 2 | 63.08 | 24.52 | 24 | 75 | 136767 | 0.0623 |
| line_101 | 1 | 75.83 | 7.97 | 24 | 76 | 191396 | 0.0873 |
| line_105 | 1 | 63.89 | 11.87 | 18 | 68.5 | 45362 | 0.0207 |
| line_109 | 1 | 60.60 | 15.39 | 25 | 60 | 136599 | 0.0623 |
| line_129 | 1 | 58.13 | 6.06 | 24 | 58 | 27485 | 0.0125 |
| line_136 | 2 | 76.56 | 11.83 | 25 | 78 | 291551 | 0.1329 |
| line_138 | 1 | 40.64 | 5.44 | 25 | 41 | 50682 | 0.0231 |
| line_142 | 2 | 63.48 | 10.79 | 25 | 68 | 70638 | 0.0322 |
| line_149 | 2 | 53.48 | 8.10 | 23 | 56 | 48985 | 0.0223 |
| line_153 | 2 | 54.96 | 9.68 | 25 | 56 | 119735 | 0.0546 |
| line_158 | 1 | 54.85 | 8.04 | 20 | 52.5 | 85662 | 0.0391 |
| line_161 | 1 | 67.72 | 8.27 | 25 | 66 | 76017 | 0.0347 |
| line_176 | 2 | 44.42 | 16.19 | 24 | 44.5 | 25060 | 0.0114 |
| line_177 | 1 | 30.16 | 7.94 | 19 | 30 | 12027 | 0.0055 |
| line_181 | 2 | 54.20 | 14.25 | 20 | 58 | 19962 | 0.0091 |
| line_189 | 2 | 62.32 | 14.20 | 22 | 64 | 9351 | 0.0043 |
| line_195 | 1 | 61.45 | 5.39 | 20 | 62.5 | 61031 | 0.0278 |
| line_208 | 1 | 53.76 | 2.60 | 25 | 54 | 38622 | 0.0176 |
| line_217 | 1 | 67.52 | 8.12 | 23 | 68 | 74816 | 0.0341 |
| line_223 | 2 | 48.73 | 5.33 | 22 | 48 | 8088 | 0.0037 |
| line_227 | 2 | 47.95 | 11.44 | 20 | 49 | 35679 | 0.0163 |
| line_228 | 1 | 65.81 | 4.17 | 21 | 66 | 58009 | 0.0264 |
| line_229 | 1 | 45.27 | 9.97 | 22 | 45 | 58098 | 0.0265 |
| line_233 | 1 | 54.24 | 9.02 | 25 | 54 | 56039 | 0.0255 |
| line_235 | 1 | 45.04 | 22.05 | 25 | 50 | 28884 | 0.0132 |
| line_237 | 2 | 60.65 | 6.19 | 17 | 61 | 295495 | 0.1347 |
| line_239 | 1 | 68.48 | 16.22 | 25 | 71 | 41365 | 0.0189 |
| line_256 | 2 | 67.67 | 12.10 | 24 | 70 | 46500 | 0.0212 |
| line_280 | 2 | 57.59 | 10.64 | 17 | 62 | 37967 | 0.0173 |
| line_287 | 2 | 41.09 | 18.80 | 23 | 44 | 107899 | 0.0492 |
| line_303 | 1 | 57.36 | NA | NA | NA | 347361 | 0.1583 |
| line_306 | 2 | 58.78 | NA | NA | NA | 71315 | 0.0325 |
| line_307 | 1 | 44.64 | NA | NA | NA | 11333 | 0.0052 |
| line_309 | 1 | 52.57 | 4.64 | 23 | 54 | 167005 | 0.0761 |
| line_310 | 2 | 22.13 | 7.82 | 24 | 21 | 51190 | 0.0233 |
| line_313 | 1 | 80.29 | NA | NA | NA | 57064 | 0.0260 |
| line_315 | 1 | 66.92 | NA | NA | NA | 39840 | 0.0182 |
| line_317 | 2 | 53.76 | 13.29 | 17 | 56 | 161118 | 0.0734 |
| line_318 | 2 | 49.36 | NA | NA | NA | 58536 | 0.0267 |
| line_320 | 2 | 65.70 | 13.07 | 23 | 69 | 9102 | 0.0041 |
| line_321 | 2 | 49.46 | 21.64 | 24 | 47.5 | 50223 | 0.0229 |
| line_324 | 1 | 48.00 | NA | NA | NA | 3943 | 0.0018 |
| line_325 | 1 | 42.61 | 4.68 | 23 | 42 | 172915 | 0.0788 |
| line_332 | 1 | 55.92 | NA | NA | NA | 83533 | 0.0381 |
| line_335 | 2 | 67.75 | NA | NA | NA | 84844 | 0.0387 |
| line_338 | 2 | 60.89 | 12.90 | 18 | 66 | 281675 | 0.1284 |
| line_340 | 2 | 59.17 | 9.01 | 24 | 60 | 33414 | 0.0152 |
| line_348 | 1 | 54.00 | 14.75 | 21 | 57 | 8446 | 0.0039 |
| line_350 | 1 | 59.25 | NA | NA | NA | 171217 | 0.0781 |
| line_352 | 2 | 46.78 | 11.52 | 23 | 48 | 194440 | 0.0886 |
| line_354 | 1 | 59.83 | 7.32 | 23 | 58 | 5064 | 0.0023 |
| line_355 | 2 | 52.45 | 8.35 | 22 | 54 | 5391 | 0.0025 |
| line_356 | 2 | 37.96 | 11.63 | 24 | 41 | 78752 | 0.0359 |
| line_357 | 1 | 58.36 | NA | NA | NA | 111899 | 0.0510 |
| line_358 | 1 | 62.67 | NA | NA | NA | 71305 | 0.0325 |
| line_359 | 1 | 56.28 | NA | NA | NA | 51370 | 0.0234 |
| line_360 | 2 | 39.91 | 5.90 | 23 | 40 | 103452 | 0.0472 |
| line_362 | 2 | 60.33 | NA | NA | NA | 76686 | 0.0350 |
| line_365 | 2 | 48.24 | 8.40 | 25 | 47 | 38529 | 0.0176 |
| line_367 | 1 | 64.15 | 8.53 | 20 | 66 | 57968 | 0.0264 |
| line_370 | 2 | 56.82 | 11.05 | 22 | 57 | 16173 | 0.0074 |
| line_371 | 1 | 39.12 | 3.11 | 25 | 40 | 8108 | 0.0037 |
| line_373 | 1 | 68.24 | 8.05 | 25 | 68 | 129299 | 0.0589 |
| line_374 | 2 | 66.04 | 8.24 | 23 | 66 | 12381 | 0.0056 |
| line_375 | 1 | 62.60 | NA | NA | NA | 48611 | 0.0222 |
| line_377 | 1 | 66.89 | 6.66 | 18 | 68 | 243461 | 0.1110 |
| line_379 | 1 | 68.08 | 16.81 | 25 | 71 | 50561 | 0.0231 |
| line_380 | 2 | 57.96 | 5.60 | 24 | 58 | 105240 | 0.0480 |
| line_381 | 1 | 41.08 | 8.83 | 25 | 40 | 169648 | 0.0773 |
| line_382 | 2 | 76.56 | 8.43 | 25 | 78 | 15422 | 0.0070 |
| line_383 | 2 | 56.84 | 10.78 | 25 | 58 | 22528 | 0.0103 |
| line_385 | 1 | 50.36 | 9.94 | 25 | 54 | 110417 | 0.0503 |
| line_386 | 1 | 54.40 | 5.06 | 25 | 55 | 111546 | 0.0509 |
| line_391 | 1 | 49.55 | 5.75 | 22 | 50 | 30426 | 0.0139 |
| line_392 | 1 | 66.29 | 7.29 | 21 | 65 | 74115 | 0.0338 |
| line_395 | 1 | 55.79 | 9.85 | 24 | 58.5 | 5285 | 0.0024 |
| line_399 | 1 | 59.26 | NA | NA | NA | 41828 | 0.0191 |
| line_405 | 2 | 50.56 | 9.35 | 25 | 50 | 174491 | 0.0795 |
| line_406 | 1 | 58.00 | 18.10 | 24 | 64 | 20083 | 0.0092 |
| line_409 | 2 | 23.92 | 9.52 | 24 | 21 | 267506 | 0.1219 |
| line_426 | 1 | 69.84 | 8.58 | 25 | 70 | 267796 | 0.1221 |
| line_427 | 1 | 52.08 | 7.40 | 24 | 50 | 40502 | 0.0185 |
| line_437 | 1 | 57.76 | 29.40 | 21 | 72 | 10576 | 0.0048 |
| line_439 | 1 | 57.80 | 12.72 | 25 | 62 | 52056 | 0.0237 |
| line_440 | 2 | 65.00 | 8.25 | 24 | 68 | 155776 | 0.0710 |
| line_441 | 2 | 65.32 | 17.65 | 25 | 70 | 19207 | 0.0088 |
| line_443 | 1 | 60.64 | 7.87 | 25 | 60 | 156133 | 0.0712 |
| line_461 | 2 | 36.96 | 8.34 | 24 | 34.5 | 27413 | 0.0125 |
| line_486 | 2 | 65.33 | 8.42 | 24 | 70 | 61682 | 0.0281 |
| line_491 | 1 | 61.13 | 8.06 | 23 | 62 | 32192 | 0.0147 |
| line_492 | 1 | 62.79 | 5.42 | 24 | 64 | 17547 | 0.0080 |
| line_502 | 1 | 69.91 | 14.05 | 23 | 72 | 144007 | 0.0656 |
| line_505 | 2 | 56.63 | 13.01 | 19 | 58 | 6193 | 0.0028 |
| line_508 | 1 | 53.60 | 8.08 | 25 | 56 | 17207 | 0.0078 |
| line_509 | 1 | 62.54 | 6.23 | 24 | 63 | 31366 | 0.0143 |
| line_513 | 2 | 51.38 | 18.29 | 24 | 52 | 33730 | 0.0154 |
| line_517 | 1 | 63.20 | 5.64 | 25 | 64 | 39249 | 0.0179 |
| line_528 | 2 | 51.08 | 14.87 | 24 | 54 | 269113 | 0.1227 |
| line_530 | 2 | 46.41 | 7.87 | 22 | 49.5 | 26680 | 0.0122 |
| line_531 | 2 | 53.61 | 19.39 | 23 | 60 | 61208 | 0.0279 |
| line_535 | 2 | 49.32 | 6.00 | 19 | 50 | 30966 | 0.0141 |
| line_551 | 2 | 59.68 | 8.98 | 25 | 59 | 158784 | 0.0724 |
| line_555 | 2 | 51.44 | 9.70 | 25 | 53 | 46876 | 0.0214 |
| line_559 | 1 | 59.76 | 6.67 | 25 | 60 | 159498 | 0.0727 |
| line_563 | 1 | 50.25 | 11.23 | 24 | 56 | 281634 | 0.1284 |
| line_566 | 1 | 65.32 | 10.60 | 25 | 64 | 109330 | 0.0498 |
| line_584 | 2 | 62.48 | 9.08 | 25 | 65 | 57830 | 0.0264 |
| line_589 | 2 | 65.96 | 15.57 | 24 | 69.5 | 5253 | 0.0024 |
| line_595 | 2 | 60.00 | 12.18 | 21 | 60 | 56380 | 0.0257 |
| line_596 | 1 | 37.94 | 11.18 | 16 | 39.5 | 5999 | 0.0027 |
| line_627 | 1 | 65.17 | 8.42 | 23 | 66 | 239238 | 0.1091 |
| line_630 | 1 | 66.08 | 13.17 | 25 | 67 | 318694 | 0.1453 |
| line_634 | 2 | 52.96 | 14.44 | 25 | 53 | 141968 | 0.0647 |
| line_639 | 2 | 75.82 | 15.23 | 22 | 79 | 52102 | 0.0238 |
| line_642 | 1 | 62.09 | 12.63 | 23 | 64 | 35075 | 0.0160 |
| line_646 | 2 | 58.00 | 17.57 | 23 | 64 | 56570 | 0.0258 |
| line_703 | 1 | 32.60 | 4.41 | 25 | 33 | 38586 | 0.0176 |
| line_705 | 2 | 63.67 | NA | NA | NA | 51922 | 0.0237 |
| line_707 | 2 | 60.64 | NA | NA | NA | 47184 | 0.0215 |
| line_712 | 2 | 45.52 | 7.84 | 25 | 47 | 42014 | 0.0192 |
| line_714 | 1 | 69.28 | 10.04 | 25 | 72 | 77196 | 0.0352 |
| line_716 | 2 | 60.00 | 14.50 | 25 | 64 | 48417 | 0.0221 |
| line_721 | 2 | 43.36 | 9.37 | 25 | 42 | 44840 | 0.0204 |
| line_727 | 2 | 39.38 | 10.82 | 21 | 41 | 18656 | 0.0085 |
| line_730 | 2 | 53.28 | 14.39 | 25 | 55 | 33330 | 0.0152 |
| line_732 | 1 | 66.76 | NA | NA | NA | 168565 | 0.0768 |
| line_737 | 2 | 53.16 | NA | NA | NA | 113123 | 0.0516 |
| line_738 | 2 | 59.88 | 7.98 | 24 | 60 | 177872 | 0.0811 |
| line_748 | 2 | 58.17 | 6.62 | 24 | 58 | 8035 | 0.0037 |
| line_757 | 1 | 29.65 | 18.25 | 20 | 29 | 19606 | 0.0089 |
| line_761 | 2 | 46.80 | 5.80 | 25 | 46 | 35037 | 0.0160 |
| line_765 | 1 | 33.96 | NA | NA | NA | 40839 | 0.0186 |
| line_774 | 1 | 64.36 | NA | NA | NA | 195136 | 0.0890 |
| line_776 | 2 | 59.75 | 6.22 | 24 | 59 | 66813 | 0.0305 |
| line_783 | 2 | 59.46 | 8.96 | 24 | 60 | 36733 | 0.0167 |
| line_786 | 2 | 53.83 | NA | NA | NA | 45760 | 0.0209 |
| line_787 | 2 | 44.92 | NA | NA | NA | 55841 | 0.0255 |
| line_790 | 2 | 49.48 | 8.66 | 23 | 52 | 29843 | 0.0136 |
| line_796 | 2 | 42.32 | 2.78 | 25 | 43 | 74641 | 0.0340 |
| line_799 | 1 | 60.00 | NA | NA | NA | 32325 | 0.0147 |
| line_801 | 2 | 69.44 | 6.34 | 25 | 72 | 134359 | 0.0613 |
| line_802 | 2 | 49.84 | 18.12 | 25 | 54 | 326828 | 0.1490 |
| line_804 | 2 | 67.24 | 13.26 | 25 | 71 | 72753 | 0.0332 |
| line_805 | 2 | 51.17 | 7.08 | 24 | 51 | 25221 | 0.0115 |
| line_808 | 1 | 52.48 | NA | NA | NA | 43379 | 0.0198 |
| line_810 | 1 | 49.12 | 15.80 | 25 | 55 | 18254 | 0.0083 |
| line_812 | 1 | 57.04 | 7.27 | 24 | 59 | 142941 | 0.0652 |
| line_818 | 2 | 60.36 | 8.65 | 25 | 62 | 71898 | 0.0328 |
| line_819 | 2 | 40.40 | 10.23 | 25 | 40 | 3750 | 0.0017 |
| line_820 | 2 | 38.92 | NA | NA | NA | 64348 | 0.0293 |
| line_821 | 2 | 72.70 | 10.42 | 23 | 74 | 126181 | 0.0575 |
| line_822 | 2 | 53.58 | NA | NA | NA | 64906 | 0.0296 |
| line_837 | 2 | 60.09 | 13.78 | 23 | 60 | 18713 | 0.0085 |
| line_843 | 1 | 53.38 | 6.96 | 24 | 56 | 23051 | 0.0105 |
| line_849 | 1 | 58.05 | 16.62 | 20 | 59.5 | 148060 | 0.0675 |
| line_850 | 2 | 44.74 | 5.43 | 23 | 44 | 5883 | 0.0027 |
| line_852 | 2 | 49.88 | NA | NA | NA | 22057 | 0.0101 |
| line_853 | 2 | 47.32 | 10.02 | 25 | 49 | 193021 | 0.0880 |
| line_855 | 2 | 54.58 | 8.32 | 24 | 56.5 | 94566 | 0.0431 |
| line_857 | 1 | 54.83 | 8.17 | 23 | 55 | 159737 | 0.0728 |
| line_859 | 2 | 60.84 | NA | NA | NA | 38944 | 0.0178 |
| line_861 | 2 | 55.67 | 14.72 | 24 | 58.5 | 79452 | 0.0362 |
| line_879 | 2 | 59.68 | 7.82 | 19 | 56 | 35422 | 0.0162 |
| line_882 | 2 | 53.54 | 19.83 | 24 | 58.5 | 25058 | 0.0114 |
| line_884 | 2 | 62.17 | 7.62 | 24 | 62 | 150768 | 0.0687 |
| line_887 | 2 | 48.04 | 10.11 | 23 | 51 | 24927 | 0.0114 |
| line_890 | 2 | 50.79 | 7.41 | 24 | 51 | 38702 | 0.0176 |
| line_892 | 2 | 51.42 | 16.77 | 24 | 58.5 | 32480 | 0.0148 |
| line_894 | 1 | 61.54 | 8.79 | 24 | 62 | 38462 | 0.0175 |
| line_897 | 2 | 49.60 | 7.80 | 20 | 52 | 18548 | 0.0085 |
| line_900 | 1 | 46.71 | 10.34 | 21 | 48 | 49074 | 0.0224 |
| line_907 | 1 | 60.44 | NA | NA | NA | 168053 | 0.0766 |
| line_908 | 1 | 47.29 | 12.74 | 21 | 45 | 46539 | 0.0212 |
| line_911 | 1 | 37.68 | NA | NA | NA | 92701 | 0.0423 |
| line_913 | 2 | 56.61 | 11.17 | 18 | 58 | 319509 | 0.1456 |

Supplementary Table 2. Genetic variation within 165 DGRP lines. ^a^Within replicate line data was available for 165 fly lines. Mean lifespan was calculated from 165 fly lines as well as the rest of the calculations; ^b^ Total genetic variance; ^c^ Variance within replicates or lines; ^d^ Total phenotypic variance ($\sigma_{G}^{2}+\sigma_{E}^{2}$); ^e^ Broad sense heritability ($\sigma_{G}^{2}/\sigma_{P}^{2}$); ^f^ Coefficient of genetic variation ($100\sigma_{G}/\text{Mean}$); ^g^ Coefficient of environmental variation ($100\sigma_{E}/\text{Mean}$);

| Mean lifespan^a^ |  |  |  |  | $CV_{G}^{f}$ | $CV_{E}^{g}$ |
| --- | --- | --- | --- | --- | --- | --- |
| 55.149 | 93.748 | 133.41 | 227.158 | 0.413 | 17.557 | 20.944 |

Supplementary Table 3. Single-SNP GWAS, genes near the top 50 SNPs; NA - not within a gene

| **SNP** | **CHR** | ***P*-value** | ***ß* coefficient** | **Within Gene** | **5'** | **5' distance [bp]** | **3'** | **3'distance [bp]** |
| --- | --- | --- | --- | --- | --- | --- | --- | --- |
| 2L_10068812_SNP | 2L | 9.41x10^-06^ | -7.41 | *CG31714* |  |  |  |  |
| 2L_10070707_SNP | 2L | 6.77x10^-06^ | -6.24 | *CG31714* |  |  |  |  |
| 2L_1632386_SNP | 2L | 5.90x10^-08^ | -5.85 | NA | *chinmo* | 18872 | *RFeSP* | 18204 |
| 2L_1632388_SNP | 2L | 3.74x10^-07^ | -5.66 | NA | *chinmo* | 18870 | *RFeSP* | 18206 |
| 2L_1696065_SNP | 2L | 2.49x10^-06^ | -7.09 | *chinmo* |  |  |  |  |
| 2L_1835028_SNP | 2L | 1.11x10^-05^ | 4.44 | NA | *c-cup* | 2472 | *wry* | 1603 |
| 2L_2279849_SNP | 2L | 2.21x10^-06^ | -11.63 | NA | *CG17242* | 10731 | *CG4271* | 5315 |
| 2L_3480710_SNP | 2L | 6.77x10^-07^ | -9.84 | NA | *CG15414* | 45 | *Thor* | 1098 |
| 2L_3746990_SNP | 2L | 1.14x10^-05^ | -9.63 | *CG10019* |  |  |  |  |
| 2L_3752571_SNP | 2L | 2.35x10^-07^ | -12.55 | *CG10019* |  |  |  |  |
| 2R_19786647_SNP | 2R | 4.66x10^-07^ | -12.29 | *Lpt* |  |  |  |  |
| 2R_4308343_SNP | 2R | 8.41x10^-06^ | -7.84 | NA | *CSN7* | 150 | *CG43296* | 3132 |
| 2R_4308355_SNP | 2R | 7.86x10^-06^ | -7.89 | NA | *CSN7* | 138 | *CG43296* | 3144 |
| 3L_11792808_SNP | 3L | 5.37x10^-06^ | -3.74 | *CG10361* |  |  |  |  |
| 3L_14778027_SNP | 3L | 3.71x10^-06^ | -10.20 | *bmm* |  |  |  |  |
| 3L_14778725_SNP | 3L | 3.50x10^-06^ | -10.17 | *bmm* |  |  |  |  |
| 3L_14780164_SNP | 3L | 4.00x10^-06^ | -10.14 | *CG13472* |  |  |  |  |
| 3L_14781414_SNP | 3L | 1.45x10^-06^ | -11.72 | *CG13472* |  |  |  |  |
| 3L_17762728_SNP | 3L | 1.13x10^-05^ | -9.58 | NA | *Adgf-A* | 5471 | *CG42815* | 21452 |
| 3L_18140585_SNP | 3L | 6.51x10^-06^ | -4.60 | NA | *CG7330* | 1438 | *gk* | 2067 |
| 3L_18810814_SNP | 3L | 4.36x10^-06^ | 4.29 | *CG14073* |  |  |  |  |
| 3L_18934159_SNP | 3L | 1.06x10^-05^ | -5.94 | *CG32204* |  |  |  |  |
| 3L_1966180_SNP | 3L | 7.14x10^-06^ | -5.81 | *CG1140* |  |  |  |  |
| 3L_4628971_SNP | 3L | 5.16x10^-06^ | -7.43 | *Rpd3* |  |  |  |  |
| 3L_5319539_SNP | 3L | 1.12x10^-05^ | -5.30 | NA | *shep* | 48496 | *lama* | 17181 |
| 3L_5373941_SNP | 3L | 7.12x10^-06^ | -11.04 | *Ir64a* |  |  |  |  |
| 3L_5636181_SNP | 3L | 2.69x10^-06^ | -9.57 | *Blimp-1* |  |  |  |  |
| 3L_8650506_SNP | 3L | 6.13x10^-06^ | -4.02 | NA | h | 18353 | *Pex7* | 6395 |
| 3L_9507749_SNP | 3L | 2.97x10^-06^ | -3.42 | *CG33700* |  |  |  |  |
| 3R_14921157_SNP | 3R | 1.15x10^-05^ | -7.33 | *ATPsyn-d* |  |  |  |  |
| 3R_15338010_SNP | 3R | 1.08x10^-05^ | -4.16 | *det* |  |  |  |  |
| 3R_15338014_SNP | 3R | 1.02x10^-05^ | -4.11 | *det* |  |  |  |  |
| 3R_15340424_SNP | 3R | 6.62x10^-06^ | -4.38 | *Dys* |  |  |  |  |
| 3R_15950064_SNP | 3R | 1.17x10^-05^ | -8.84 | NA | *Gr92a* | 2066 | *CG5023* | 45095 |
| 3R_18577501_SNP | 3R | 5.63x10^-06^ | -7.22 | *CG7023* |  |  |  |  |
| 3R_19071977_SNP | 3R | 5.64x10^-07^ | -5.62 | *CG4467* |  |  |  |  |
| 3R_20944700_SNP | 3R | 9.10x10^-06^ | -5.99 | *CG31510* |  |  |  |  |
| 3R_21259405_SNP | 3R | 7.92x10^-06^ | -9.58 | *Fur1* |  |  |  |  |
| 3R_21913681_SNP | 3R | 1.04x10^-05^ | -5.28 | *dys* |  |  |  |  |
| 3R_23482833_SNP | 3R | 9.26x10^-06^ | -3.51 | NA | *Mlc1* | 452 | *tau* | 115 |
| 3R_24748071_SNP | 3R | 1.19x10^-05^ | -3.92 | *Doa* |  |  |  |  |
| 3R_25189263_SNP | 3R | 1.05x10^-05^ | -6.41 | NA | *Cnx99A* | 43898 | *Ptp99A* | 13725 |
| 3R_25562159_SNP | 3R | 5.27x10^-06^ | -4.18 | *CG7601* |  |  |  |  |
| 3R_25921654_SNP | 3R | 1.04x10^-05^ | -8.93 | *sima* |  |  |  |  |
| 3R_25921693_SNP | 3R | 9.79x10^-06^ | -8.94 | *sima* |  |  |  |  |
| 3R_25921696_SNP | 3R | 9.79x10^-06^ | -8.94 | *sima* |  |  |  |  |
| 3R_8922024_SNP | 3R | 1.19x10^-05^ | -7.43 | *timeout* |  |  |  |  |
| X_20940365_SNP | X | 4.95x10^-06^ | -14.60 | *bves* |  |  |  |  |
| X_604933_SNP | X | 8.31x10^-06^ | -3.53 | *sdk* |  |  |  |  |
| X_9282626_SNP | X | 2.78x10^-06^ | 7.06 | *mgl* |  |  |  |  |

Supplementary Table 4. Top 30 genes, gene-based analysis. *Empirical *p*-values, based on 1,000,000 permutations

| **FlyBaseGene ID** | **Gene Symbol** | **Chr** | **N SNPs** | **Gene-based*p*-value^*^** |
| --- | --- | --- | --- | --- |
| FBgn0036603 | *CG13062* | 3L | 22 | 7.10x10^-05^ |
| FBgn0036870 | *CG14095* | 3L | 5 | 2.97x10^-04^ |
| FBgn0037985 | *ssp5* | 3R | 18 | 3.29x10^-04^ |
| FBgn0051956 | *pgant4* | 2L | 74 | 5.08x10^-04^ |
| FBgn0039462 | *CG14252* | 3R | 62 | 5.32x10^-04^ |
| FBgn0087005 | *rtp* | 3R | 2 | 5.47x10^-04^ |
| FBgn0029843 | *Nep1* | X | 161 | 6.22x10^-04^ |
| FBgn0039075 | *CG4393* | 3R | 194 | 7.01x10^-04^ |
| FBgn0037156 | *CG11523* | 3L | 5 | 9.02x10^-04^ |
| FBgn0016120 | *ATPsyn-d* | 3R | 10 | 9.63x10^-04^ |
| FBgn0051928 | *CG31928* | 2L | 33 | 9.73x10^-04^ |
| FBgn0036008 | *CG3408* | 3L | 68 | 9.87x10^-04^ |
| FBgn0050154 | *CG30154* | 2R | 21 | 9.99x10^-04^ |
| FBgn0036208 | *CG10361* | 3L | 70 | 1.02x10^-03^ |
| FBgn0263004 | *CG43312* | 3L | 2 | 1.03x10^-03^ |
| FBgn0044324 | *Chro* | 3L | 26 | 1.08x10^-03^ |
| FBgn0031596 | *CG15429* | 2L | 46 | 1.24x10^-03^ |
| FBgn0262818 | *CG43189* | 2R | 3 | 1.26x10^-03^ |
| FBgn0025638 | *Roc1a* | X | 5 | 1.51x10^-03^ |
| FBgn0036165 | *chrb* | 3L | 137 | 1.58x10^-03^ |
| FBgn0035011 | *CG13589* | 2R | 17 | 1.64x10^-03^ |
| FBgn0037307 | *Tim17a2* | 3R | 9 | 1.65x10^-03^ |
| FBgn0039385 | *CG5913* | 3R | 19 | 1.70x10^-03^ |
| FBgn0033769 | *CG8768* | 2R | 34 | 1.82x10^-03^ |
| FBgn0039890 | *CG2316* | 4 | 25 | 1.98x10^-03^ |
| FBgn0037960 | *mthl5* | 3R | 48 | 2.11x10^-03^ |
| FBgn0051998 | *CG31998* | 4 | 16 | 2.11x10^-03^ |
| FBgn0032217 | *CG4972* | 2L | 52 | 2.32x10^-03^ |
| FBgn0260003 | *Dys* | 3R | 3104 | 2.40x10^-03^ |
| FBgn0031601 | *Dim1* | 2L | 24 | 2.44x10^-03^ |

Supplementary Table 5. Top 30 genes, gene-based analysis (genes±5kb). *Empirical *p*-values, based on 1,000,000 permutations

| **FlyBaseGene ID** | **Gene Symbol** | **Chr** | **N SNPs** | **Gene-based*p*-value^*^** |
| --- | --- | --- | --- | --- |
| FBgn0029843 | *Nep1* | X | 245 | 6.61x10^-04^ |
| FBgn0035827 | *CG14252* | 3L | 337 | 7.57x10^-04^ |
| FBgn0262275 | *mir-2280* | 2L | 312 | 8.62x10^-04^ |
| FBgn0015300 | *Ssl* | 2R | 224 | 9.17x10^-04^ |
| FBgn0086075 | *CR34704* | 3L | 316 | 1.06x10^-03^ |
| FBgn0052783 | *CG32783* | X | 20 | 1.09x10^-03^ |
| FBgn0031367 | *c-cup* | 2L | 349 | 1.18x10^-03^ |
| FBgn0053703 | *CG33703* | 3L | 369 | 1.32x10^-03^ |
| FBgn0010408 | *RpS9* | 3L | 375 | 1.37x10^-03^ |
| FBgn0017556 | Prosα4T2 | 2R | 240 | 1.42x10^-03^ |
| FBgn0029501 | *Crtp* | 2R | 253 | 1.43x10^-03^ |
| FBgn0051998 | *CG31998* | 4 | 60 | 1.59x10^-03^ |
| FBgn0039890 | *CG2316* | 4 | 66 | 1.61x10^-03^ |
| FBgn0053702 | *CG33702* | 3L | 371 | 1.75x10^-03^ |
| FBgn0061188 | *Yu* | 2R | 213 | 1.78x10^-03^ |
| FBgn0262988 | *CG43296* | 2R | 195 | 1.95x10^-03^ |
| FBgn0025387 | *CG12184* | X | 93 | 2.03x10^-03^ |
| FBgn0053701 | *CR33701* | 3L | 390 | 2.27x10^-03^ |
| FBgn0035012 | *CG13590* | 2R | 199 | 2.31x10^-03^ |
| FBgn0035011 | *CG13589* | 2R | 188 | 2.44x10^-03^ |
| FBgn0263344 | *CR43425* | 4 | 28 | 2.47x10^-03^ |
| FBgn0036008 | *CG3408* | 3L | 409 | 2.53x10^-03^ |
| FBgn0053700 | *CG33700* | 3L | 701 | 2.80x10^-03^ |
| FBgn0035281 | *Cpr62Bc* | 3L | 377 | 2.82x10^-03^ |
| FBgn0039889 | *Arl4* | 4 | 49 | 2.98x10^-03^ |
| FBgn0260003 | *Dys* | 3R | 3271 | 3.30x10^-03^ |
| FBgn0044324 | *Chro* | 3L | 80 | 3.34x10^-03^ |
| FBgn0053978 | *CG33978* | 4 | 91 | 3.45x10^-03^ |
| FBgn0037202 | *Ssl1* | 3L | 69 | 3.72x10^-03^ |
| FBgn0052786 | *CG32786* | X | 9 | 3.73x10^-03^ |

Supplementary Table 6. Genes belonging to the IIS and TOR pathways

| IIS pathway genes | *14-3-3epsilon* (*FBgn0020238*), *Akt1* (*FBgn0010379*), *B4* (*FBgn0023407*), *chico* (*FBgn0024248*), *dock* (*FBgn0010583*), *foxo* (*FBgn0038197*), *hpo* (*FBgn0261456*), *Ilp1* (*FBgn0044051*), *Ilp2* (*FBgn0036046*), *Ilp3* (*FBgn0044050*), *Ilp4* (*FBgn0044049*), *Ilp5* (*FBgn0044048*), *Ilp6* (*FBgn0044047*), *Ilp7* (*FBgn0044046*), *Ilp8* (*FBgn0036690*), *Impl2* (*FBgn0001257*), *InR* (*FBgn0013984*), *Lnk* (*FBgn0028717*), *melt* (*FBgn0023001*), *Phlpp* (*FBgn0032749*), *Pi3K21B* (*FBgn0020622*), *Pi3K92E* (*FBgn0015279*), *Pten* (*FBgn0026379*), *S6KII* (*FBgn0262866*), *sgg* (*FBgn0003371*), *step* (*FBgn0086779*), *wdb* (*FBgn0027492*), *Pdk1* (*FBgn0020386*), *B4* (*FBgn0023407*) |
| --- | --- |
| TOR pathway genes | *Atg1* (*FBgn0260945*), *chrb* (*FBgn0036165*), *dm* (*FBgn0262656*), *Dredd* (*FBgn0020381*), *eIF-4B* (*FBgn0020660*), *eIF-4E* (*FBgn0015218*), *eIF4G* (*FBgn0023213*), *gig* (*FBgn0005198*), *HLH106* (*FBgn0261283*), *L* (*FBgn0001332*), *lkb1* (*FBgn0038167*), *Lst8* (*FBgn0264691*), *Mipp2* (*FBgn0026060*), *Mo25* (*FBgn0017572*), *par-1* (*FBgn0260934*), *path* (*FBgn0036007*), *pgc* (*FBgn0016053*), *pico* (*FBgn0261811*), *Pka-C1* (*FBgn0000273*), *RagA* (*FBgn0037647*), *RagC* (*FBgn0033272*), *raptor* (*FBgn0029840*), *Rheb* (*FBgn0041191*), *rictor* (*FBgn0031006*), *RpS6* (*FBgn0261592*), *S6k* (*FBgn0015806*), *scyl* (*FBgn0041094*), *Sesn* (*FBgn0034897*), *Sik2* (*FBgn0025625*), *Sin1* (*FBgn0033935*), *slif* (*FBgn0037203*), *SNF1A* (*FBgn0023169*), *Thor* (*FBgn0261560*), *Tor* (*FBgn0021796*), *tor* (*FBgn0003733*), *Crtc* (*FBgn0036746*), *Tsc1* (*FBgn0026317*), *Tif-IA* (*FBgn0032988*) |

Supplementary Table 7. Ranking and *p*-values of the top 20 GO categories

| **GO ID** | **Go category** | ***p*-value** | **Number of genes** |
| --- | --- | --- | --- |
| GO:0046692 | sperm competition | 2.21x10^-04^ | 22 |
| GO:0022626 | cytosolic ribosome | 2.68x10^-04^ | 4 |
| GO:0046527 | glucosyltransferase activity | 3.04x10^-04^ | 11 |
| GO:0019318 | hexose metabolic process | 4.44x10^-04^ | 63 |
| GO:0006006 | glucose metabolic process | 4.87x10^-04^ | 43 |
| GO:0035074 | pupation | 5.51x10^-04^ | 5 |
| GO:0006007 | glucose catabolic process | 5.65x10^-04^ | 33 |
| GO:0006433 | prolyl-tRNA aminoacylation | 5.76x10^-04^ | 2 |
| GO:0004827 | proline-tRNA ligase activity | 5.76x10^-04^ | 2 |
| GO:0006096 | glycolysis | 8.47x10^-04^ | 25 |
| GO:0043564 | Ku70:Ku80 complex | 9.67x10^-04^ | 3 |
| GO:0003684 | damaged DNA binding | 1.10x10^-03^ | 23 |
| GO:0004197 | cysteine-type endopeptidase activity | 1.10x10^-03^ | 28 |
| GO:0000090 | mitotic anaphase | 1.25x10^-03^ | 17 |
| GO:0046365 | monosaccharide catabolic process | 1.29x10^-03^ | 34 |
| GO:0019320 | hexose catabolic process | 1.29x10^-03^ | 34 |
| GO:0045254 | pyruvate dehydrogenase complex | 1.42x10^-03^ | 4 |
| GO:0006085 | acetyl-CoA biosynthetic process | 2.11x10^-03^ | 4 |
| GO:0005996 | monosaccharide metabolic process | 2.27x10^-03^ | 67 |
| GO:0008534 | oxidized purine nucleobase lesion DNA N-glycosylase activity | 2.81x10^-03^ | 2 |

Supplementary Table 8. Ranking and *p*-values of the top 20 GO categories (genes±5kb)

| **GO ID** | **Go category** | ***p*-value** | **Number of genes** |
| --- | --- | --- | --- |
| GO:0007442 | Hindgut morphogenesis | 7.27x10^-04^ | 19 |
| GO:0016485 | Protein processing | 8.38x10^-04^ | 696 |
| GO:0051604 | Protein maturation | 9.21x10^-04^ | 699 |
| GO:0004165 | dodecenoyl-CoA delta-isomerase activity | 9.47x10^-04^ | 5 |
| GO:0035079 | Polytene chromosome puffing | 1.12x10^-03^ | 8 |
| GO:0007350 | Blastoderm segmentation | 1.21x10^-03^ | 21 |
| GO:0000090 | Mitotic anaphase | 1.24x10^-03^ | 17 |
| GO:0006508 | proteolysis | 1.44x10^-03^ | 671 |
| GO:0043564 | Ku70:Ku80 complex | 1.57x10^-03^ | 3 |
| GO:0070011 | Peptidase activity acting on L-amino acid peptides | 1.61x10^-03^ | 596 |
| GO:0004177 | Aminopeptidase activity | 1.81x10^-03^ | 39 |
| GO:0042600 | chorion | 2.14x10^-03^ | 38 |
| GO:0006426 | glycyl-tRNA aminoacylation | 2.39x10^-03^ | 2 |
| GO:0004820 | glycine-tRNA ligase activity | 2.39x10^-03^ | 2 |
| GO:0008233 | Peptidase activity | 2.41x10^-03^ | 602 |
| GO:0004822 | isoleucine-tRNA ligase activity | 2.42x10^-03^ | 2 |
| GO:0006428 | isoleucyl-tRNA aminoacylation | 2.42x10^-03^ | 2 |
| GO:0008527 | Taste receptor activity | 2.44x10^-03^ | 62 |
| GO:0045254 | Pyruvate dehydrogenase complex | 2.48x10^-03^ | 4 |
| GO:0004175 | endopeptidase activity | 2.85x10^-03^ | 445 |

Supplementary Table 9. Polygenic score (permuted vs. original lifespan). SEM- standard error of the mean

| **p threshold** | **Mean *R^2^***  **(Lifespan)** | **SEM**  **(Lifespan)** | **Mean *R^2^***  **(permuted lifespan)** | **SEM**  **(permuted lifespan)** | **One-sided**  **t-test *p*-value** |
| --- | --- | --- | --- | --- | --- |
| ≤1.0 | 0.042 | 0.0030 | 0.019 | 0.0019 | 9.83x10^-10^ |
| ≤0.9 | 0.042 | 0.0031 | 0.019 | 0.0019 | 9.18x10^-10^ |
| ≤0.8 | 0.042 | 0.0031 | 0.019 | 0.0019 | 8.39x10^-10^ |
| ≤0.7 | 0.043 | 0.0031 | 0.019 | 0.0019 | 7.05x10^-10^ |
| ≤0.6 | 0.043 | 0.0031 | 0.020 | 0.0019 | 6.56x10^-10^ |
| ≤0.5 | 0.043 | 0.0031 | 0.020 | 0.0019 | 5.88x10^-10^ |
| ≤0.2 | 0.043 | 0.0031 | 0.019 | 0.0019 | 2.19x10^-10^ |
| ≤0.1 | 0.044 | 0.0031 | 0.019 | 0.0019 | 5.26x10^-11^ |
| ≤0.05 | 0.044 | 0.0031 | 0.019 | 0.0019 | 1.76x10^-11^ |
| ≤0.01 | 0.047 | 0.0032 | 0.018 | 0.0019 | 4.57x10^-13^ |
| ≤0.005 | 0.047 | 0.0031 | 0.019 | 0.0002 | 7.23x10^-13^ |
| ≤0.001 | 0.047 | 0.0032 | 0.019 | 0.0019 | 1.57x10^-12^ |
| ≤0.0005 | 0.046 | 0.0031 | 0.020 | 0.0021 | 4.79x10^-11^ |
| ≤0.0001 | 0.042 | 0.0030 | 0.020 | 0.0020 | 4.02x10^-09^ |

References

1. Mackay TF, Richards S, Stone EA, Barbadilla A, Ayroles JF, Zhu D*, et al.* The *Drosophilamelanogaster* Genetic Reference Panel.Nature.2012;482:173-178.

2. Huang W, Massouras A, Inoue Y, Peiffer J, Ramia M, Tarone A*, et al.* Natural variation in genome architecture among 205 *Drosophilamelanogaster* Genetic Reference Panel lines.Genome Res.2014.

3. Arya GH, Weber AL, Wang P, Magwire MM, Negron YL, Mackay TF*, et al.* Natural variation, functional pleiotropy and transcriptional contexts of odorant binding protein genes in *Drosophilamelanogaster*.Genetics.2010;186:1475-1485.

4. Vieira C, Pasyukova EG, Zeng ZB, Hackett JB, Lyman RF, Mackay TF. Genotype-environment interaction for quantitative trait loci affecting life span in *Drosophila melanogaster*.Genetics.2000;154:213-227.

5. Barrett JC, Fry B, Maller J, Daly MJ. Haploview: analysis and visualization of LD and haplotype maps.Bioinformatics.2005;21:263-265.

6. Larkin MA, Blackshields G, Brown NP, Chenna R, McGettigan PA, McWilliam H*, et al.* Clustal W and Clustal X version 2.0.Bioinformatics.2007;23:2947-2948.
